# The Effects of Different Exercise Interventions on Patients with Subjective Cognitive Decline: A Systematic Review and Network Meta-Analysis

**DOI:** 10.14283/jpad.2024.65

**Published:** 2024-03-26

**Authors:** R. Chen, B. Zhao, J. Huang, M. Zhang, Y. Wang, J. Fu, H. Liang, Hongrui Zhan

**Affiliations:** 1 Department of Rehabilitation Medicine, The Fifth Affiliated Hospital of Sun Yat-sen University, Zhuhai City, Guangdong Province, China; 2 College of Rehabilitation Medicine, Fujian University of Traditional Chinese Medicine, Fuzhou City, Fujian Province, China

**Keywords:** Subjective cognitive decline, cognitive function, exercise, network meta-analysis

## Abstract

**Background and Objective:**

Exercise is a promising non-pharmacological therapy for subjective cognitive decline, but it is unclear which type of exercise is most effective. The objective was to assess the comparative effects and ranks of all exercise-based interventions on cognitive function in patients with subjective cognitive decline (SCD).

**Method:**

In this network meta-analysis, Online databases for Web of Science, PubMed, Embase, Medline, Cochrane Library and PsycINFO were searched from inception to April 30, 2023. The included studies are randomized controlled trials assessing the efficacy of exercise interventions for individuals with SCD. The primary outcome measure is memory, while secondary outcome measures encompass executive function, attention, verbal fluency, and global cognitive function. Represented using Standardized Mean Differences (SMDs) along with their 95% Confidence Intervals (CIs). Bias assessment was conducted in accordance with the ‘Cochrane Risk of Bias Assessment Tool, 2nd Edition’ (RoB 2). Pairwise meta-analysis was carried out using the ‘meta-analysis’ module within STATA 14.0, and network meta-analysis was performed using the ‘mvmeta’ and ‘network’ packages available in STATA 14.0. Registration number CRD42023289687.

**Result:**

This study included a total of 11 randomized controlled trials, encompassing 1,166 patients. Mind-body exercise was found to be efficacious in enhancing or sustaining memory (SMD: 0.58, 95%CI: 0.06 ∼ 1.10) and executive function (SMD: 0.41, 95%CI: 0.09 ∼ 0.73) in individuals with subjective cognitive decline. Furthermore, mind-body exercise exhibited the highest probability of being the most effective measures for improving or preventing the decline in memory (surface under cumulative ranking curve (SUCRA) value: 90.4) and executive function (SUCRA value: 91.8). The second-ranked moderate-intensity aerobic exercise has also shown a positive effect on the improvement of executive function in patients with subjective cognitive decline (SMD: 0.23, 95%CI: 0.03 ∼ 0.43, SUCRA value: 68.2). However, we did not observe a significant effectiveness of exercise interventions on verbal fluency, attention, and overall cognitive function in subjective cognitive decline.

**Conclusion:**

Mind-body exercise may potentially be the optimal strategies for enhancing memory and executive function in individuals with subjective cognitive decline. Additionally, moderate-intensity aerobic exercise has shown a modest positive effect on executive function in subjective cognitive decline. When resources permit, practical application of these findings may be considered. Nevertheless, further support for the conclusions of this study is warranted through larger sample sizes and well-designed multicenter trials.

**Electronic Supplementary Material:**

Supplementary material is available in the online version of this article at 10.14283/jpad.2024.65.

## Introduction

Subjective Cognitive Decline (SCD) refers to a decline in subjective memory or cognitive function without apparent cognitive impairments in objective cognitive assessments and without impairment in activities of daily living (1). SCD stands as an intermediate state between normal aging and Mild Cognitive Impairment (MCI), acknowledged as one of the earliest phases and initial cognitive alterations in the onset of Alzheimer’s disease (AD) (2), carrying a heightened risk of progression to MCI or AD (3). Epidemiological investigations indicate that among individuals aged 50 and older, the prevalence of SCD is 26.6% (4). SCD is associated with a 1.73-fold increased risk of progression to MCI in older adults and a 1.9-fold increased risk of progression to AD (5).

In the early stages of the AD progression, the brain can functionally compensate for neuropathological changes, thereby enabling individuals with SCD to maintain objective cognitive scores within the normal range on neuropsychological tests (6). Recent research (7) reports that individuals with SCD, despite not demonstrating significant declines in objective neuropsychological assessments, exhibit subtle impairments in global cognitive function, memory, executive function, and language abilities when compared to healthy controls. Notably, the most pronounced deterioration is observed within the domain of memory (7). While SCD does not meet the diagnostic criteria for MCI, it does share analogous patterns of cerebral alterations with patients diagnosed with MCI and those suffering from dementia attributed to AD (8).

Neuroimaging research (9) reveals that AD has undergone progressive neurofunctional deterioration and incurs irreversible cognitive impairment. Hence, the genuine promise in treating AD may lie in early intervention (10). As a preclinical stage of AD and MCI, SCD potentially represents a critical therapeutic window for slowing or preventing cognitive deterioration (11). Early intervention holds the promise of reversing cognitive decline and mitigating the risk of developing AD(11). Given the limited efficacy of pharmacological interventions in enhancing cognitive function in patients with SCD, along with the potential for adverse effects, non-pharmacological interventions have garnered considerable attention for their impact on cognitive rehabilitation in SCD (12).

Exercise, as one of the non-pharmacological interventions, can modulate neuronal electrical activity associated with cognitive function, enhance brain structural plasticity (13), stimulate the generation of new neurons and synapses related to learning and memory (14, 15), promote the secretion of neurotrophic factors such as brain-derived neurotrophic factor (BDNF) and insulin-like growth factor-1 (IGF-1) (16, 17), to subsequently enhance cognitive function. Exercise has a positive impact on the cognitive function of individuals with SCD (18, 19). The «Physical Activity Guidelines for Americans» (20)also explicitly recommend that older individuals can enhance their cognitive function and reduce the risk of developing AD through regular physical activity.

Previous pertinent research shows that diverse forms of exercise, such as aerobic exercise (AE), resistance exercise (RE) and mind-body exercise (MBE), may potentially exert cognitive enhancement effects through the modulation of distinct molecular mechanisms outlined above, resulting in variable magnitudes of impact (e.g., MBE: standard mean difference (SMD) =0.38 0.52 0.72 (18, 21, 22), AE: SMD= 0.14 0.24 0.31 (18, 21, 23), RE: SMD=0.22 0.29 0.39 (18, 21, 24)). Hence, the choice of exercise modality constitutes a pivotal consideration for clinical professionals when devising exercise prescriptions aimed at preventing or mitigating cognitive decline (25).

Nonetheless, the absence of clinical studies concurrently comparing various types of exercise interventions, coupled with the scarcity of available direct evidence (head-to-head randomized clinical trials) within the literature incorporated in traditional pairwise meta-analyses, poses a formidable challenge in assessing the comparative efficacy of different exercise therapies. Therefore, the most efficacious exercise treatment modality for preventing or mitigating cognitive decline in SCD patients remains unclear at present. This ambiguity makes it challenging for healthcare professionals to draw definitive conclusions regarding the “most effective”exercise types and to formulate exercise intervention measures that are most effective in treating SCD patients.

Network Meta-Analysis (NMA) serves as a robust quantitative framework that amalgamates both direct and indirect evidence stemming from clinical trial networks (26). It facilitates the assessment of the effectiveness of different clinical interventions based on clinical evidence, effectively surmounting the limitations inherent in traditional meta-analyses (27). Furthermore, NMA also enables the ranking of intervention measures, yielding a hierarchy of all exercise therapies. Understanding which exercise selection is deemed the “most effective” can assist physicians in making clinical decisions, informing clinical practice, and integrating the optimal type of exercise into the patient’s rehabilitation objectives.

## Methods

This systematic review was conducted following the PRISMA (28) and the PRISMA Extension Statement for Reporting of Systematic Reviews Incorporating Network Meta-analyses (29). The protocol for this study was registered with PROSPERO (registration number CRD42023289687).

### Search strategy

Online databases for Web of Science, PubMed, Embase, Medline, Cochrane Library and PsycINFO were searched from inception to April 30, 2023. Referring to the study by Huang et al (30), the search strategy was initially devised for PubMed and subsequently adapted for other databases. The details of the retrieval strategy formulated for PubMed can be found in the appendix 1. The retrieval process was independently conducted by two researchers. In case of any disagreements, consensus was reached through discussions between the two researchers.

### Study selection

The literature search records will be uploaded to Endnote X9, and a deduplication process will be conducted within the software. In the first stage, two researchers (BZ and JH) independently conducted a screening of the titles and abstracts of relevant articles, followed by a meticulous full-text assessment in strict accordance with the inclusion and exclusion criteria. Any discrepancies were resolved through consensus reached through discussions between the two researchers or, if necessary, adjudicated by a third researcher.

The detailed inclusion criteria as follows: (1) The study design must adhere to a randomized controlled trial (RCT) methodology. (2) The Participants must be diagnosed with SCD (meet the SCD conceptual framework proposed by Jessen et al. in 2014(1) and China AD Preclinical Alliance(11)). (3) Intervention measures may encompass any form of exercise training. (4) The control group must fall into one of the following categories: standard care, health education, blank control (without administering any treatment or implementing any specific interventions) or treatment as usual. (5) The study must report at least one of the following outcomes: global cognitive function, memory function, executive function, attention, and verbal fluency. (6) The research must be documented in the English language. The SCD conceptual framework as follows: 1) subjective decline of memory rather than other domains of cognition; 2) onset of SCD within the last 5 years; 3) worries associated with SCD; and 4) worse self-perceived memory than others in the same age group; 5) absence of objective clinical impairment of MCI(the Mini-Mental State Examination (MMSE), the Montreal Cognitive Assessment (MoCA) or the modified Mini Mental Status examination (3MS) are within the normal range after years of education correction, and have not been clinically diagnosed as MCI as determined by clinical doctors).

The detailed exclusion criteria are as follows: 1) Studies specifically examining cognitive impairment in patients with various types of cancer, Parkinson’s disease, Huntington’s disease, epilepsy, multiple sclerosis, Psychiatric illnesses (e.g., major depression, schizophrenia and bipolar disorder) (these diseases, aside from being associated with cognitive dysfunction, typically exhibit diverse pathological changes; consequently, they may interfere the effects of exercise on cognitive function). 2) Significant cerebrovascular lesions (e.g., evident cerebral infarction or evident cerebral hemorrhage) (11). 3) Studies specifically investigating the effects of acute exercise (the effects generated after a single instance of acute exercise). 4) Comprehensive intervention measures in which exercise is not the primary component (e.g., exercise combined with cognitive training or exercise combined with physical therapy).

Outcomes: The core symptom of SCD is a decline in memory function (31), followed by impairments in executive function, attention and verbal fluency domains (32). Therefore, we used memory function as the primary outcome measure and analyzed executive function, attention, verbal fluency and global cognitive function as secondary outcome measures. The assessment scales with different outcome measures, arranged in order based on their frequency of use and psychometric characteristics, are listed sequentially in Appendix 2. When utilizing multiple instruments to assess a particular cognitive dimension, we select the most appropriate tool based on predefined criteria.

### Data extraction

Two researchers (JF and HL) independently established data extraction forms according to the Cochrane Handbook’s guidelines (33), then proceeded to independently extract data, cross-referencing their findings. The extracted data encompassed general information (authors, publication year), participant characteristics (population, gender, average age), study features (number of patients, intervention measures, control measures, intervention duration, frequency, duration of each session), and outcomes (means, standard deviations (SDs), respective measurement tools). In instances where relevant statistical metrics were reported incompletely, we employed estimation methods for mean and SD based on sample size, median, range, and p-values, in accordance with the Cochrane Handbook guidelines (33). Additionally, we initiated email correspondence with the authors to procure any missing or incomplete data.

During the data extraction process, in order to assess the efficacy of various types of exercise interventions, we categorized exercise interventions following the American College of Sports Medicine Exercise Testing and Prescription Guidelines (20) and previous systematic reviews (34, 35). These categories included Moderate-Intensity aerobic exercise (MI, such as walking, running, and cycling, etc), High-Intensity aerobic exercise (HI, such as Boxing and Track and Field, etc), Resistance exercise (RE, aimed at increasing muscle strength, e.g., using elastic tubes, elastic bands, and weight machines), Multicomponent exercise (ME, combining two or more types of exercise, such as MI combine RE and balance training) and Mind-body exercise (MBE, emphasizing the integration of movement with breathing, mindfulness and memory, including practices like baduanjin, yoga, and mind-motor exercise). To investigate the moderating variables of exercise effects, we categorized and coded exercise frequency, intensity, duration per session, and intervention duration (Appendix 3).

### Risk of bias assessment

Two researchers (MZ and JH) independently conducted methodological quality assessments of the included RCTs using the “Cochrane Risk of Bias Assessment Tool, 2nd Edition” (RoB 2) (36). Divergences were resolved through consensus discussions, and in cases where differences couldn’t be reconciled, consultation with a senior researcher was sought. Studies were categorized into low, high, or some concerns of bias based on the following criteria: Randomization process; Deviations from intended interventions; Missing outcome data; Measurement of the outcome; Selection of the reported result.

### Data synthesis and statistical analysis

#### Pairwise meta-analyses

We utilized the “meta-analysis” module in Stata 14.0 (Verson 14.0; StataCorp, College Station, TX, USA) to perform pairwise analyses for all direct comparisons, thereby elucidating the effects of various exercise interventions compared to the control group individually. Depending on the magnitude of heterogeneity in the data, we employed the random-effects model (I-V heterogeneity method) or the fixed-effects model (inverse variance method) to calculate the standardized mean difference (SMD) and its corresponding 95% confidence interval (95% CI) for the differences in scores before and after the intervention. Using the I^2^ statistic to estimate the proportion of total variance attributed to heterogeneity between studies in each pairwise comparison.

#### Network meta-analysis

Network meta-analyses (NMA) were conducted using the “mvmeta” (37, 38) and “network” (39, 40) packages in Stata 14.0 (Verson 14.0; StataCorp, College Station, TX, USA), based on a frequentist analysis framework, for both primary and secondary outcome measures. NMA integrates the results from individual studies, and each treatment effect of the intervention/control group can be obtained through direct or indirect comparison (41, 42). When there is no direct connection between two treatment arms, the results are based on indirect evidence (41, 42). To visualize the network geometry and connectivity of nodes, we created network diagrams for each cognitive outcome measure. Each node represents an intervention, and the connecting lines between two nodes represent one or more direct comparisons (43). The size of each node is proportional to the number of participants receiving that intervention, with larger nodes indicating a higher number of participants who received the intervention (43). The thickness of the connecting lines is related to the number of studies that directly compared these two interventions, with thicker lines representing a greater number of studies (43).

We initially employ an inconsistency model for global inconsistency analysis. A p-value below 0.05 in the inconsistency test indicates the presence of global inconsistency (44). The local inconsistency analysis was conducted using the node-splitting method. The presence of local incongruity is signified when the p-value of the incongruity test falls below 0.05 (44). When evidence closed loops are present in the network diagram, we use the loop-specific method for loop inconsistency analysis. When the incongruity factor approaches zero, it signifies the concordance between two sources of evidence (44).

Fitting with multivariate random-effects (restricted maximum likelihood estimation) meta-analysis model in the framework of frequentism. This model takes into consideration the heterogeneity between studies caused by clinical and other factors, providing more conservative confidence intervals (CI) for the combined point estimates, in order to account for the interrelation of effect sizes among trials involving more than two groups (44, 45). After combining direct and/or indirect comparisons for any pair of interventions, we computed pooled effect sizes represented as SMDs with corresponding 95% CIs. The effect sizes were categorized as small (SMD <0.40), moderate (0.40 ≤ SMD ≤ 0.70), or large (SMD >0.70) following the Cochrane handbook guidelines(33).

To rank exercise interventions, we utilized a parameter-guided bootstrapping procedure with 10,000 resamples to assess the effectiveness of each intervention and calculated the Surface Under the Cumulative Ranking curve (SUCRA) values. SUCRA is a precise estimation of the cumulative ranking probability for the top i treatments. For each treatment j among the n compared treatments, the cumulative probability of treatment j being ranked within the top i is calculated using the following formula: $\text{SUCRA}_{j}=(\sum\nolimits_{\rm{i}=1}^{\rm{n}-1}\ cum_{j,i})/(\rm{n}-1)$ (46). The range of SUCRA values spans from 0% to 100%, and the closer the value approaches 100%, the higher the likelihood that the intervention is more effective.

#### Regression and sensitivity analysis

To further explore the sources of heterogeneity and inconsistency, we conducted a Meta-regression analysis on the primary outcome measures, using the frequency, intensity, duration, and duration of exercise interventions as covariates.

A sensitivity analysis was conducted by excluding RCTs with at least one domain assessed as high risk of bias in pairwise meta-analyses, aiming to explore the robustness of the study outcomes.

#### Small study effects

Egger’s test was employed to assess the presence of small study effects. Additionally, funnel plots were generated for visual inspection of publication bias for each comparison of outcomes (47).

## Results

### Literature search and selection

**Figure 1:**
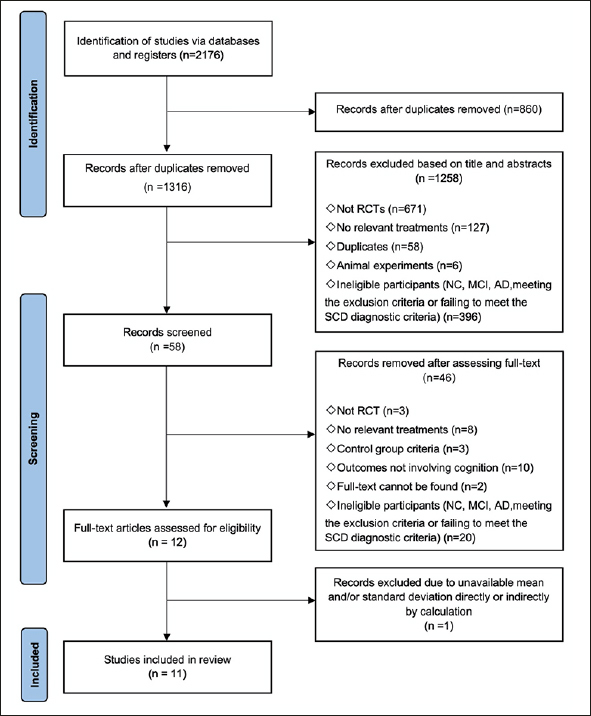
Flow of studies in the review Note: RCT: Randomized controlled trial, NC: Normal cognitive, SCD: Subjective cognitive decline, MCI: Mild cognitive impairment, AD: Alzheimer’s disease.

After deduplication, a total of 1316 records were identified. Among these records, 58 were considered potentially relevant following the initial screening of titles and abstracts. Following the application of inclusion and exclusion criteria, a total of 11 randomized controlled trials, comprising 1166 participants, were included in the network meta-analysis (Fig 1). The appendix 4 provide detailed characteristics of the studies included in the analysis, and the appendix 5 contain all citations of the studies included in the NMA.

### Characteristics of included studies

Among the included studies, the sample sizes ranged from 31 to 415, with a mean age of 62.87 years. Eight studies (1079 participants) investigated the impact of exercise on memory function (48–55), eight studies (921 participants) examined the influence of exercise on executive function (48, 49, 51–54, 56, 57), while three studies (573 participants) explored the effects of exercise on attention (48, 51, 53), three studies (610 participants) investigated the influence of exercise on verbal fluency (49, 53, 58), and six studies (729 participants) assessed the impact of exercise on global cognitive function (48, 49, 52, 53, 56, 57). There are a total of five intervention categories, which include Moderate-Intensity aerobic exercise (MI, N = 8) (49–53, 55, 57, 58), High-Intensity aerobic exercise (HI, N = 2) (52, 56), Resistance exercise (RE, N = 2) (48, 53), Multicomponent exercise (ME, N = 3) (50, 53, 56), and Mind-body exercise (MBE, N = 3) (52, 54, 58) (Appendix 4, Appendix 5).

### Risk of bias

The summary of bias risk can be found in the supplementary materials. All included studies were RCTs, but 31% of the trials did not adequately report the implementation methods of randomization. Out of the 11 trials, 6 trials (54.5%) were rated as having a low risk of bias. For these 6 trials, a significant proportion of “Some concerns” (36.4%) arose due to the lack of detailed descriptions regarding group concealment or the handling of missing outcome data. One trial (9.1%) received a high risk of bias assessment due to the absence of researcher blinding (Appendix 6).

### Pairwise meta-analyses

Appendix 7 presents the outcomes of pairwise meta-analysis and estimates of heterogeneity. In brief, exercise interventions were found to be more efficacious than control groups in the domains of memory (Combine SMD=0.20, 95 % CI: 0.07∼0.34, I^2^=33.8%), executive function (−0.15, 95 % CI: −0.29 ∼ −0.01, I^2^=25.0%), and verbal fluency (−0.21, 95%CI: −0.35 ∼ −0.06, I^2^=9.3 %) in SCD. Nevertheless, exercise interventions did not exhibit significant differences in improving global cognitive function (*P*=0.72, I^2^=0.0%) and attention (*P*=0.88, I^2^=0.0%) in SCD compared to the control groups.

### Network meta-analysis

**Figure 2:**
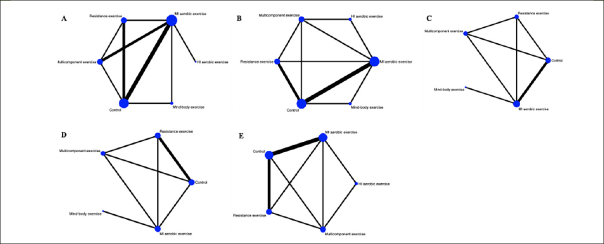
Network plot of available treatment comparisons Note: Each node represents an intervention, and the connecting lines between two nodes represent one or more direct comparisons. The size of each node is weighted based on the number of participants receiving that intervention, while the thickness of the connecting lines is weighted based on the number of studies directly comparing those two interventions. A: memory, B: executive function, C: verbal fluency, D: attention, E: global cognitive function. MI aerobic exercise: Moderate-Intensity aerobic exercise, HI aerobic exercise: High-Intensity aerobic exercise.

**Figure 3:**
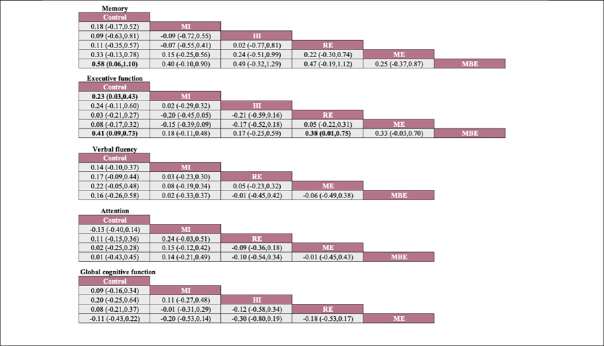
Network meta-analysis of effectiveness comparison Note: Each cell displays the SMD with a 95% confidence interval. For any cell, a negative SMD favors interventions in the upper-left corner, while a positive SMD favors interventions in the lower-right corner. Significant results are highlighted in bold. MI: Moderate-Intensity aerobic exercise, HI: High -Intensity aerobic exercise, RE: Resistance exercise, ME: Multicomponent exercise, MBE: Mind-body exercise.

**Figure 4:**
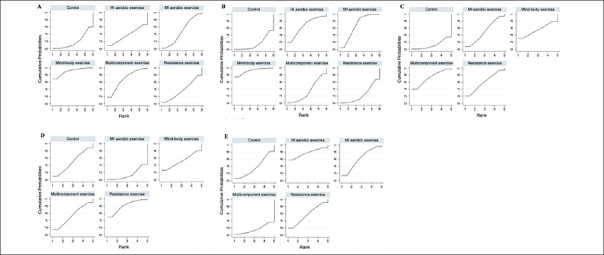
Cumulative ranking probability plot Note: A: memory, B: executive function, C: verbal fluency, D: attention, E: global cognitive function. MI aerobic exercise: Moderate-Intensity aerobic exercise, HI aerobic exercise: High-Intensity aerobic exercise.

A total of five different interventions and control arms were included, comprising 1166 patients with SCD in our network meta-analysis. The inconsistency test based on network analysis showed no statistically significant differences in global inconsistency (memory: *P*=0.07, executive function: *P*=0.39, verbal fluency: *P*=0.10, attention: *P*=0.61, global cognitive function: *P*=0.22), detailed results are provided in the appendix 8. When evaluating closed-loop networks, no statistically significant differences in inconsistency between direct and indirect outcomes were observed. Detailed results can be found in the appendix 8. The network diagram (Fig 2) displays the weights of all available comparisons included in this network meta-analysis. Comparative effects between exercise interventions can be found in the league table (Fig 3). Cumulative probability plots for different exercise interventions and the ranking of SUCRAs are presented in Fig 4 and Table 1, respectively.

**Table 1:** The memory and executive function rankings for different types of exercise.

**Intervention**	**Memory function**			**Executive function**		
	**SUCRA**	**Mean rank**	**P (%)**	**SUCRA**	**Mean rank**	**P (%)**
Control	19.4	5.0	0.1	15.5	5.2	0.0
MI	47.1	3.6	7.8	68.2	2.6	19.7
HI	37.3	4.1	0.8	67.1	2.6	4.9
RE	37.3	4.1	3.4	23.5	4.8	0.4
ME	68.5	2.6	17.3	33.8	4.3	1.0
MBE	90.4	1.5	70.6	91.8	1.4	74.0

Notes: MI = Moderate-Intensity aerobic exercise; HI = High-Intensity aerobic exercise; RE = Resistance exercise; ME=Multicomponent exercise; MBE = Mind-body exercise.

The network meta-analysis results indicate a significant impact of mind-body exercise on memory function in SCD patients when compared to the control group (combined SMD: 0.58, 95%CI: 0.06 ∼ 1.10). There were no significant differences between different types of exercises. Mind-body exercise (MBE) had the highest probability (70.6%) of being the most effective exercise modality, with a SUCRA value of 90.4. Followed by multicomponent exercise (ME, SUCRA=68.5, P=17.3%), moderate-intensity aerobic exercise (MI, SUCRA=47.1, P=7.8%), resistance exercise (RE, SUCRA=37.3, P=3.4%), and high-intensity aerobic exercise (HI, SUCRA=37.3, P=0.8%). Refer to Fig 3, Fig 4 and table 1 for details.

For executive function in SCD patients, MBE (combined SMD: 0.41, 95%CI: 0.09 ∼ 0.73) and moderate-intensity aerobic exercise (combined SMD: 0.23, 95%CI: 0.03 ∼ 0.43) demonstrated significantly greater improvements compared to the control group. Furthermore, mind-body exercise significantly outperformed resistance exercise in improving executive function (combined SMD: 0.38, 95%CI: 0.01 ∼ 0.75). MBE has the highest probability (74.0%) of being the most effective exercise type for preserving executive function, with a SUCRA value of 91.8. Following that are MI (SUCRA=68.2, P=19.7%), HI (SUCRA=67.1, P=4.9%), ME (SUCRA=33.8, P=1%), and RE (SUCRA=23.5, P=0.4%).

Refer to Fig 3, Fig 4 and table 1 for details.

However, we did not observe significant differences between different types of exercise interventions compared to the control group or in comparisons between different types of exercise interventions in the remaining cognitive dimensions. Network plots, cumulative ranking plots, and SUCRA values for exercise interventions in the remaining cognitive dimensions are presented in Fig 2, Fig 3, Fig 4, as well as Appendix 8.

### Regression and sensitivity analysis

Meta-regression analyses were separately conducted for different types of exercise interventions (primarily focusing on the main outcome measure: memory), with exercise intervention frequency, intensity, duration per session, and intervention duration were included as covariates. The results show that the intensity of each session serves as a moderating factor affecting the effectiveness of exercise interventions on memory in individuals with SCD (see the appendix 9 for details).

We excluded the study (56) with a high risk of bias in at least one domain, which encompassed cognitive domains such as global cognitive function and executive function. Sensitivity analysis revealed that the outcomes of the interventions remained unchanged (see the appendix 10 for details).

### Small study effects

Overall, we did not find compelling evidence of small study effects among the outcomes. The points on the funnel plots for each study domain are visually symmetrically distributed around the mean estimated treatment effect (see the appendix 11 for details). The p-values for Egger’s test were as follows: 0.39 for memory, 0.84 for executive function, 0.14 for verbal fluency, 0.87 for attention, and 0.36 for global cognition (see the appendix 11 for details).

## Discussion

This systematic review and network meta-analysis on exercise interventions for patients with Subjective Cognitive Decline (SCD) included data from 11 clinical trials involving a total of 1166 participants. To our knowledge, this review is the first network meta-analysis aimed at exploring the relative efficacy of different types of exercise on cognitive function in SCD. Our study results corroborate the beneficial impact of exercise interventions on cognitive function in SCD and highlight Mind-Body Exercise (MBE) as the most promising exercise therapy for attenuating memory and executive function decline in SCD patients.

Over the past few decades, the beneficial effects of MBE on cognitive function have gradually become a research focus. MBE represents a multimodal form of exercise that emphasizes the harmonious integration of mind, body, and spirit (59). In addition to aiding in balance control, flexibility, and muscle strength, MBE also places emphasis on mental focus, procedural memory, physical equilibrium, and relaxation (60). Compared to aerobic and resistance exercises that focus primarily on cardiovascular fitness and strength, MBE integrate movement sequences with breath control and attention regulation. Additionally, they have been shown to increase oxygenated hemoglobin levels in the prefrontal cortex (61). The combination of these physical and neural resources can offer an explanation for the observed differences in the effects of exercise therapy.

Furthermore, the combined nature of ME involving two or more modalities presents challenges in ensuring that each exercise component meets optimal durations, frequencies, and intensities during training. This can lead to a diminished practical efficacy of ME in real-world applications. It is worth noting that we observed exercise intensity as a moderating variable influencing the magnitude of exercise effects, with very high-intensity exercise not necessarily resulting in improved memory function for SCD individuals. MBE typically involves slow-paced and low-intensity activities, making it a more suitable option for older adults compared to other forms of exercise (62, 63). This may potentially serve as another explanation for why MBE demonstrates the greatest potential in mitigating cognitive decline in SCD. Researchers should also take these factors into consideration in future studies.

Recent research findings have supported the potential relationship between physical activity and neural changes, indicating that MBE can modulate brain structures (64) and induce alterations in brain neural activity and functional connectivity (64, 65), including regions such as the hippocampus (66) and prefrontal cortex (64), which play a crucial role in cognitive function. Currently, consistent observations indicate reduced structural integrity in the hippocampus and prefrontal gray matter in individuals with SCD (67–70), as well as a decrease in the functional connectivity between the hippocampus and prefrontal regions (71, 72). The hippocampus and prefrontal cortex are the core regions responsible for memory and executive function processes, respectively (73, 74). The modulation of brain structure, neural activity, and functional connectivity by MBE may serve as the foundation for the beneficial effects of such exercise on memory and executive function in SCD.

MBE is also highly likely to synergize its benefits in enhancing memory and executive function through other neurobiological mechanisms that may initiate favorable biochemical alterations. For instance, MBE may enhance cognitive function by upregulating levels of brain-derived neurotrophic factor (BDNF) in the plasma, which is a crucial mediator of central nervous system neuroplasticity (75). Additionally, it may also exert beneficial effects on the brain and cognition by modulating inflammatory cytokines (levels of which are associated with cognitive impairment (76–78)) such as tumor necrosis factor-alpha (TNF-α), interleukin-6 (IL-6), IL-10, and IL-1β, with studies (79–81) reporting that, levels of inflammatory cytokines are associated with cognitive impairment. These mechanisms contribute to its potential to enhance cognitive function. Overall, our research findings suggest that MBE may have unexpected benefits for memory and executive function in SCD patients, consistent with previous meta-analytic (82) results.

For executive function, while the efficacy is lower compared to MBE, we also observed a facilitative effect on executive function from moderate-intensity aerobic exercise (MI). Prior research (83) has indicated that executive function is the cognitive domain most sensitive to the beneficial effects of aerobic exercise. Potential underlying mechanisms may involve aerobic exercise’s modulation of cerebral vascular health, enhancement of cerebral blood flow, and regional cortical thickness, which induce cortical activation in the prefrontal subregions (84–86). In previous research (87–89), it has been observed that the intensity of aerobic exercise selectively impacts executive function, with high-intensity aerobic exercise appearing to have less pronounced effects on the improvement of executive function, which aligns with our findings. They propose that the positive impact of aerobic exercise on executive function follows an inverted U-shaped curve, increasing from no intensity to moderate intensity and then declining with further increases in intensity (87–89). Additionally, the release of various neurochemical substances related to cognitive function (e.g., catecholamines, cortisol, BDNF) induced by exercise also depends on factors such as exercise intensity (90).

Consistent with the study by Coelho et al. (91), we cannot provide evidence suggesting a significant enhancement of executive function in SCD through combined exercise. This may be related to the substantial disparities in exercise intensity and frequency used in some studies compared to the activity standards recommended for older adults. The American College of Sports Medicine (20) recommends that older adults should engage in at least 5 days of moderate-intensity exercise for 30–60 minutes per day each week. However, within the articles included in this meta-analysis, the highest intervention frequency observed for studies focusing on executive function was 3 times, and it only involved one study (52). The insufficient exercise dosage may potentially attenuate the positive effects of ME.

It is noteworthy that within our network meta-analysis, no significant efficacy of exercise on attention, verbal fluency, and global cognitive function in individuals with SCD was observed. In the preclinical stage of Alzheimer’s disease, SCD typically exhibits the earliest cognitive impairment in the domain of memory, followed by executive functions (92). We speculate that the relatively limited effects of exercise on attention and verbal fluency cognitive functions may be attributed to the less severe impairment of these cognitive domains in individuals with SCD. However, Venegas-Sanabria et al. (93), when assessing the impact of exercise on individuals with Alzheimer’s disease or mild cognitive impairment, also found that exercise did not yield significant beneficial effects on verbal fluency and attention. The emergence of such results may be related to the insensitivity of the domains of attention and language fluency to exercise, as cognitive domains exhibit varying levels of sensitivity to exercise (e.g., aerobic exercise having the most pronounced therapeutic effect on executive functions (83)). Interestingly, in our conventional pairwise meta-analysis, we observed a positive effect of exercise on verbal fluency in SCD. However, this finding was based on the inclusion of only one study (53). Given the limited number of clinical trials within the studies included in the analysis that have investigated the impact of exercise on attention and verbal fluency in SCD, the findings warrant cautious interpretation and necessitate further experiments in the future to augment the required evidence.

In addition, one of the diagnostic criteria for SCD is the absence of objectively demonstrable clinical impairment associated with MCI. Based on this diagnostic criterion, the neurobehavioral scores for global cognitive function in the SCD patients included in the analysis were within the normal range, and baseline data demonstrated preserved cognitive performance. This suggests that there is only minimal decline in global cognitive function, leaving limited room for exercise to improve global cognitive function in SCD. It has been reported that exercise interventions require a sufficiently long duration to exert an impact on specific cognitive domains (94, 95). The majority of studies included in the analysis had a duration of 6 months or less, which impedes the long-term investigation of exercise effects on attention, verbal fluency, and global cognitive function in SCD. As for the mechanisms involved in this, more clinical trials are needed to fill this gap.

We anticipate that our research findings will hold significant implications for both clinical decision-making and scientific investigation. In the context of clinical rehabilitation, MBE deserves heightened attention and can be recommended as an adjunct therapy for SCD patients due to its significant benefits in improving memory and executive function. MI can also be considered as a routine non-pharmacological treatment for SCD patients to enhance their executive function. Certainly, future clinical trials should undertake more investigations into elucidating the mechanisms by which exercise influences SCD cognitive functions to explain the reasons behind the benefits it confers. For clinical researchers, regular updates to NMAs on this topic are indispensable as new data continues to emerge.

While our meta-analysis demonstrated the beneficial impact of exercise on SCD cognition, there are still some limitations to consider. Firstly, the number of studies included in our review was limited, and to validate our findings, large-scale trials will be needed in the future. Secondly, only a small number of studies have reported long-term follow-up data after the conclusion of interventions. Consequently, we extracted data only from the included studies for the period after the interventions were completed, resulting the sustained benefits of exercise on various aspects of SCD cognition unverified. Furthermore, among the included studies, only one study (53) employed a multi-arm design, directly comparing the effects of different types of exercise on SCD cognition. Many effect size estimates were reliant on indirect comparisons. Considering that evidence from direct comparisons is more robust than indirect comparisons, it is imperative to conduct more multi-arm designed studies in the future.

Our NMA also has other limitations, such as a lack of exploration into the most effective exercise dosages (intensity, frequency, session duration, and length of intervention). This is another critical factor influencing rehabilitation outcomes. The dose-response relationship of exercise interventions on SCD cognitive function needs further investigation. In addition, due to the unavailability of complete data, we did not conduct subgroup analysis. Future research endeavors may explore whether different forms of exercise are beneficial for distinct types of SCD (e.g., the impact of aerobic or resistance exercise on cognitive function in SCD with cardiovascular risk). This would be an intriguing topic, as research (96) suggests a novel direction in dementia prevention by offering diverse intervention measures tailored to individual prevention needs, varying with lifestyle factors and identified risks of cognitive decline. Lastly, our NMA only included articles written in English, which could potentially result in the omission of information from eligible research reports conducted in other languages.

## Conclusion

This network meta-analysis has synthesized the existing evidence from clinical studies, offering clinical professionals and researchers some important findings regarding exercise therapy. Our research reveals that among individuals experiencing SCD, MBE emerges as the most effective exercise modality in slowing down the decline in memory and executive function. Additionally, MI demonstrates a favorable capacity for improving executive function in SCD patients. Nevertheless, in light of the limitations of our aforementioned meta-analysis and the paucity of studies in the current literature, the results should be interpreted with caution. To bolster the findings of this NMA, further investigation is warranted, including well-designed, large-scale, multi-arm, multicenter trials.

### Electronic Supplementary Material

Supplementary material, approximately 374 KB.

## Notes

*Ethical standards:* Not required.
