# Supplementary material for: The Effects of Different Exercise Interventions on Patients with Subjective Cognitive Decline: A Systematic Review and Network Meta-Analysis
Source: J Prev Alzheimers Dis. 2024 Mar 26;11(3):620–31. doi: 10.14283/jpad.2024.65 (PMC11060994; doi:10.14283/jpad.2024.65)
Supplement: Supplementary file 1 — Supplementary material, approximately 374 KB. [file 42414_2024_65_MOESM1_ESM.docx]

Content

[Appendix 1. Search strategy 2](#_Toc148384601)

[Appendix 2. Measurement tools 3](#_Toc148384602)

[Appendix 3. Exercise parameters 4](#_Toc148384603)

[Appendix 4. Study characteristics 5](#_Toc148384604)

[Appendix 5. Citations of studies. 8](#_Toc148384605)

[Appendix 6. Risk of bias 9](#_Toc148384606)

[Appendix 7. Pairwise meta-analyses 11](#_Toc148384607)

[Appendix 8. Network meta-analysis 16](#_Toc148384608)

[Appendix 9. Regression analysis 26](#_Toc148384609)

[Appendix 10. Sensitivity analysis 27](#_Toc148384610)

[Appendix 11. Small study effects 29](#_Toc148384611)

# Appendix 1. Search strategy

Table 1. Search strategy in PubMed

| Step | Search strategy |
| --- | --- |
| #1 | Search (((((((subjective cognitive decline[Title/Abstract]) OR subjective cognitive impairment[Title/Abstract]) OR subjective memory decline[Title/Abstract]) OR subjective memory impairment[Title/Abstract]) OR cognitive complaints[Title/Abstract]) OR memory complaints[Title/Abstract]) |
| #2 | Search (exercise[MeSH Terms]) OR (((((((((((exercises[Title/Abstract]) OR physical activit*[Title/Abstract]) OR training*[Title/Abstract]) OR danc*[Title/Abstract]) OR yoga[Title/Abstract]) OR taichi[Title/Abstract]) OR wuqinxi[Title/Abstract]) OR baduanjin[Title/Abstract]) OR yijinjing[Title/Abstract]) OR qigong[Title/Abstract]) |
| #3 | Search ((randomized controlled trial [pt] OR controlled clinical trial [pt] OR randomized [tiab] OR placebo [tiab] OR clinical trials as topic [mesh: noexp] OR randomly [tiab] OR trial [ti]) NOT (animals [mh] NOT humans [mh])) |
| #4 | #4 #1 AND #2 AND #3 |

# Appendix 2. Measurement tools

Table 2. The measurement tools for different outcome measures

| Memory function | Auditory verbal learning test (AVLT); Wechsler Memory Scale-Revised-Logical memory (WMS-R LM); Hopkins Verbal Learning Test (HVLT); Memory Complaints Scale (MCS); Word list total immediate recall; Frequency of Forgetting (FOF); Paired Associates; Cambridge Brain Sciences (CBS)-memory. |
| --- | --- |
| Executive cognition | Trial mark test (TMT-B, TMT-A); Stroop color word test (SCWT);  Indirect Digit Span; Digit-Symbol Coding Test; Monkey Ladder. |
| Verbal fluency | Category Fluency Test; Phonemic verbal fluency; Delis-Kaplin Function Battery. |
| Attention | Digit Span Forward; Direct Digit Span. |
| Global cognition | Mini-Mental State Examination (MMSE); Alzheimer’s Disease Assessment Scale-Cognitive Subscale (ADAS-Cog); Cambridge cognitive test (CAMCOG); Cambridge Brain Sciences (CBS) battery. |

# Appendix 3. Exercise parameters

Table 3. Re-encoding of exercise parameters^[1, 2]^

| Frequency | |
| --- | --- |
| Low | 1-2 times/week |
| Moderate | 3-4 times/week |
| High | ≥5 times/week |
| Duration per session |  |
| Short | <40 minutes |
| Moderate | 40-59 minutes |
| long | ≥60 minutes |
| Length of intervention |  |
| Short | <13 weeks (3 months) |
| Moderate | 14-25 weeks (3 months-6 months) |
| long | ≥25 weeks (≥6 months) |
| Intensity | |
| Low | 1. < 40%HRR/ < 60%HR max/ 60% V̇O2max /  2. ≤11RPE  3. < 50% of 1 RM |
| Moderate | 1. 40%-60%HRR/ 60%-85% HR max/60–80% V̇O_2_max  2. 12-13RPE  3. 60–80% of 1 RM |
| High | 1. 60-90%HRR/ >85% HR max/80% V̇O_2_max  2. ≥14RPE  3. 80–100% of 1 RM |

References:

[1] Chen F T, Etnier J L, Chan K H, et al. Effects of Exercise Training Interventions on Executive Function in Older Adults: A Systematic Review and Meta-Analysis [J]. Sports Med, 2020, 50(8): 1451-67.

[2] Wang S, Yin H, Wang X, et al. Efficacy of Different Types of Exercises on Global Cognition in Adults with Mild Cognitive Impairment: A Network Meta-Analysis [J]. Aging Clin Exp Res, 2019, 31(10): 1391-400.

# Appendix 4. Study characteristics

Table 4. detailed characteristics of studies

| **study** | **Participants (exercise vs control)** | | | | | | | | **interventions** | | | | **comparator** | **Outcomes**  **(GC=global cognition, EF=executive function, Mem=memory, Att=attention, Ver=verbal fluency)** |
| --- | --- | --- | --- | --- | --- | --- | --- | --- | --- | --- | --- | --- | --- | --- |
|  | **Population** | **Sample size** | **Age** | **Female/male** | **hypertension** | **Diabetes** | **Heart disease** | **BMI** | **type** | **duration** | **frequency** | **Length** |  |  |
| Busse, A.L.2008 | SCD | 14 vs 17 | 73.3±6.4 vs 70.4±3.6 | 15/2 vs 8/6 | 7 vs 10 | 4 vs 3 | 1 vs 1 | NA | Resistance training | 60 | 2 | 36 | Usual care | **GC** (CAMCOG) 89.20±23.56 vs 84.50±20.27  **Mem** (MCS) 8.20±2.37 vs 8.20±2.14  **EF** (Indirect Digit Span) 4.50±0.77 vs 4.20±0.65  **Att** (Direct Digit Span) 4.60±6.22 vs 4.90±2.67 |
| Lautenschlager, N.T.2008 | SCD | 85 vs 85  (SCD:37 vs 31) | 68.6±8.7 vs 68.7±8.5 | NA | 30 vs 30 | NA | 8 vs 10 | NA | Moderate-Intensity aerobic exercise | 50 | 3 | 24 | Health Education | **GC** (ADAS-Cog) 0.53±6.7 vs 0.69±5.17  **Mem** (Word list total immediate recall)  1.51±8.2 vs 2.08±9.5  **EF** (Digit-Symbol Coding Test)  3.35±12.46 vs 5.66±16.67  **Ver** (Delis-Kaplin) 3.99±23.67 vs -0.8±20.89 |
| Zuniga, K.E.2016 | SCD | 90 vs 89 | 66.4±5.7 | 117/62 | NA | NA | NA | NA | Moderate-Intensity aerobic walking | 60 | 3 | 24 | Multicomponent exercise (muscle toning exercises, balance, and yoga) | **Mem** (FOF) 48.83±1.05 vs 49.41±1.05 |
| Boa Sorte Silva, N.C.2018 | SCD | 63 vs 64 | 67.6±7.5 vs 67.4±7.2 | 44/19 vs 46/18 | 36 vs 32 | 7 vs 5 | 5 vs 4 | 29±4.1 vs 29.7±6.2 | Mind-body exercise | 60 | 3 | 24 | Multicomponent exercise (balance, range of motion and breathing) | **Ver** (phonemic verbal fluency) -0.05±1.03 vs -0.03±1.00 |
| Boa Sorte Silva, N.C.2020 |  |  |  |  |  |  |  |  |  |  |  |  |  | **EF** (Monkey Ladder) 0.23±1.43 vs 0.29±1.40  **Att** (Digit Span Forward) 0.33±2.82 vs -0.06±2.76  **Mem** (Paired Associates) 0.48±2.22 vs 0.01±2.16 |
| Boa Sorte Silva, N.C.2021 | SCD | 65 vs 63 | 71.7±6.3 vs 70.4±7.1 | 32/33 vs 29/34 | 51±78.5 vs 49±77.8 | 11±16.9 vs 13±20.6 | NA | 29.3 ±5.8 vs 30.3 ±6.5 | High-Intensity aerobic exercise | 60 | 3 | 24 | Moderate-Intensity aerobic exercise | **GC** (CBS) -0.03±0.89 vs 0.04±0.83  **Mem** (CBS-memory) -0.004±1.33 vs 0.11±1.27  **EF** (TMT-A) -0.06±0.32 vs -0.07±0.32 |
| Ramnath, U. 2021 | SCD | 23 vs 22 | 70.8±4.52 vs 74.14±5.8 | NA | 18 vs 15 | 10 vs 4 | NA | 70.8 ±4.52 vs 74.14 ±5.8 | High-Intensity aerobic exercise (bowling, Boxing, Track and Field, Table Tennis, Soccer, Beach Volleyball) | 60 | 2 | 12 | Multicomponent exercise（strength training and proprioceptive exercises） | **GC (MMSE)** 27.3±2.49 vs 25.3±2.49  **EF (SCWT)** 1533.3±261.28 vs 1613.8±261.58 |
| Makino, T.2021 | SCD | 104 vs 102 vs 104 vs 105 | 72.3±4.6 vs 72.3±4.8 vs 72.6±4.5 vs 72.1±4.6 | 49/55 vs 49/52 vs 43/61 vs 54/51 | NA | NA | NA | NA | (1) Moderate-Intensity aerobic exercise (2) Resistance exercise  (3) combined training (MI+RE) | 40 | 2 | 26 | received lectures about health promotion | **GC** (MMSE**)**  0.75±2.34 vs 0.78±2.32 vs 0.54±2.24 vs 0.61±2.35  **Mem** (WMS-R, LM II)  2.74±4.79 vs 1.87±4.64 vs 1.88±4.58 vs 1.36±4.81  **Att** (Digit Span Forward)  -0.27±1.41 vs 0.07±1.29 vs -0.07±1.30 vs -0.11±1.41  **Ver** (Category Fluency Test)  0.25±4.16 vs 0.35±4.12 vs 0.16±4.06 vs 1.29±4.18  **EF** (TMT B)  -4.13±46.31 vs 1.29±45.14 vs 0.04±44.75 vs 4.26±46.22 |
| Su, H. 2021  (PP) | SCD | 32 vs 33 | 64.40±6.57 vs 65.37±6.31 | 19/16 vs 17/18 | NA | NA | NA | NA | Baduanjin exercise and Health Education | 60 | 5 | 12 | Health Education | **Mem** (AVLT)  40.83±5.39 vs 36.26±5.53  **EF** (TMT(A+B))  134.54 ± 25.71 vs 152.54 ± 24.71 |
| Stroehlein, J.K.2021 | SCD | 23 vs 19 | 67.87±4.7 vs 67.89±3.9 | 13/10 vs 10/9 | 12 vs 7 | 3 vs 2 | 4 vs 3 | 26.3 ± 3.4 vs 26.2 ± 3.9 | Golf  (Moderate) | 60 | 3 | 22 | keep their lifestyle and sports activities unchanged | **GC** (ADAS-Cog)  6.82±2.67 vs 6.58±2.97  **EF** (TMT B)  47.58±12.57 vs 52.66±29.03 |
| Chou,C.C. 2022 | SCD | 33 vs 33 | 69.3±6.5 vs 69.8±7.6 | all female | 7 vs 6 | NA | NA | 23.6 ± 2.6 vs 24.9 ± 3.1 | Moderate-Intensity aerobic walking | 30 | 5 | 24 | routine care and a manual on healthy living | **Mem** (HVLT)  25.50±4.50 vs 24.00±4.50 |

*Note*: NA: not available; BMI: Body mass index; MMSE: Mini-Mental State Examination; MoCA: The Montreal cognitive assessment; ADAS-Cog: Alzheimer’s Disease Assessment Scale-Cognitive Subscale (ADAS-Cog,)

# Appendix 5. Citations of studies.

[1] Busse A L, Filho W J, Magaldi R M, et al. Effects of Resistance Training Exercise on Cognitive Performance in Elderly Individuals with Memory Impairment: Results of a Controlled Trial [J]. Einstein, 2008, 6(4).

[2] Lautenschlager N T, Cox K L, Flicker L, et al. Effect of Physical Activity on Cognitive Function in Older Adults at Risk for Alzheimer Disease: A Randomized Trial [J]. Jama, 2008, 300(9): 1027.

[3] Zuniga K E, Mackenzie M J, Kramer A, et al. Subjective Memory Impairment and Well-Being in Community-Dwelling Older Adults [J]. Psychogeriatrics, 2016, 16(1): 20-6.

[4] Boa Sorte Silva N C, Gill D P, Gregory M A, et al. Multiple-Modality Exercise and Mind-Motor Training to Improve Mobility in Older Adults: A Randomized Controlled Trial [J]. Exp Gerontol, 2018, 103: 17-26.

[5] Boa Sorte Silva N C, Nagamatsu L S, Gill D P, et al. Memory Function and Brain Functional Connectivity Adaptations Following Multiple-Modality Exercise and Mind-Motor Training in Older Adults at Risk of Dementia: An Exploratory Sub-Study [J]. Front Aging Neurosci, 2020, 12: 22.

[6] Boa Sorte Silva N C, Petrella A F M, Christopher N, et al. The Benefits of High-Intensity Interval Training on Cognition and Blood Pressure in Older Adults with Hypertension and Subjective Cognitive Decline: Results from the Heart & Mind Study [J]. Front Aging Neurosci, 2021, 13: 643809.

[7] Makino T, Umegaki H, Ando M, et al. Effects of Aerobic, Resistance, or Combined Exercise Training among Older Adults with Subjective Memory Complaints: A Randomized Controlled Trial [J]. J Alzheimers Dis, 2021, 82(2): 701-17.

[8] Ramnath U, Rauch L, Lambert E V, et al. Efficacy of Interactive Video Gaming in Older Adults with Memory Complaints: A Cluster-Randomized Exercise Intervention [J]. PLoS One, 2021, 16(5): e0252016.

[9] Stroehlein J K, Vieluf S, Zimmer P, et al. Learning to Play Golf for Elderly People with Subjective Memory Complaints: Feasibility of a Single-Blinded Randomized Pilot Trial [J]. BMC Neurology, 2021, 21(1).

[10] Su H, Wang H, Meng L. The Effects of Baduanjin Exercise on the Subjective Memory Complaint of Older Adults: A Randomized Controlled Trial [J]. Medicine (Baltimore), 2021, 100(30): e25442.

[11] Chou C C, Chien L Y, Lin M F, et al. Effects of Aerobic Walking on Memory, Subjective Cognitive Complaints, and Brain-Derived Neurotrophic Factor among Older Hypertensive Women [J]. Biol Res Nurs, 2022, 24(4): 484-92.

# Appendix 6. Risk of bias

Fig 1. Risk of bias for all included studies


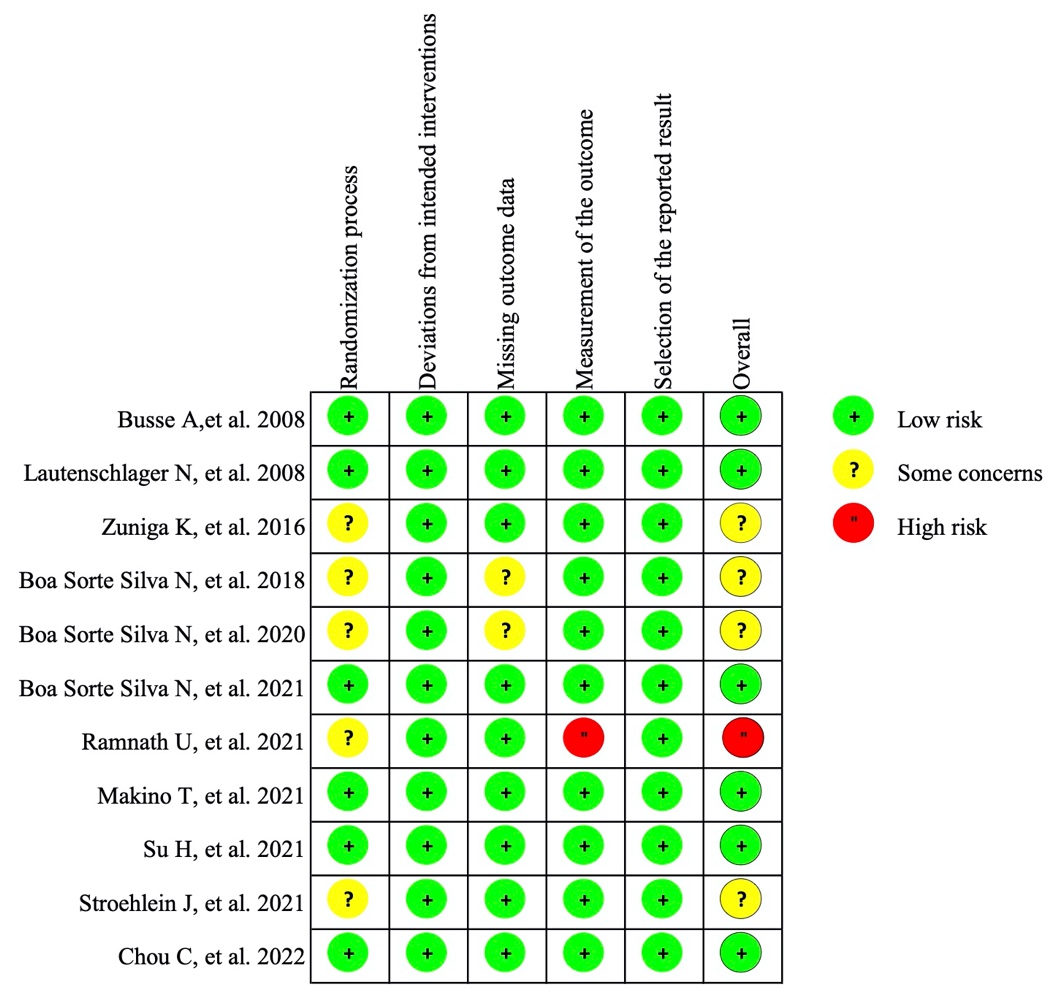


Fig 2. Risk of bias for each study

Table 5. Risk of bias assessment

# Appendix 7. Pairwise meta-analyses

**7.1 pairwise analysis for memory**

Fig 3. Forest plot of pairwise meta-analysis for memory

*Note*: MI: Moderate-Intensity aerobic exercise, HI: High-Intensity aerobic exercise, RE: Resistance exercise, Mul: Multicomponent exercise, Min: Mind-body exercise.

Table 6. Pairwise meta-analysis of effectiveness for memory


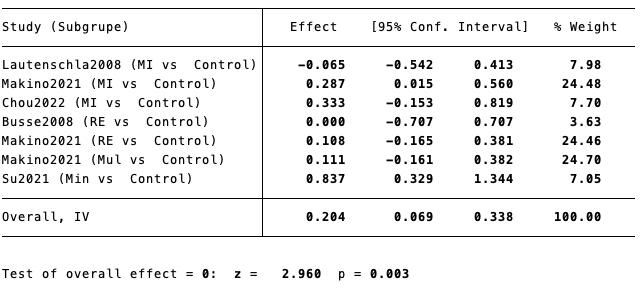


*Note*: MI: Moderate-Intensity aerobic exercise, HI: High-Intensity aerobic exercise, RE: Resistance exercise, Mul: Multicomponent exercise, Min: Mind-body exercise.

**7.2 pairwise analysis for executive function**

Fig 4. Forest plot of pairwise meta-analysis for executive function

*Note*: MI: Moderate-Intensity aerobic exercise, RE: Resistance exercise, Mul: Multicomponent exercise, Min: Mind-body exercise.

Table 7. Pairwise meta-analysis of effectiveness for executive function


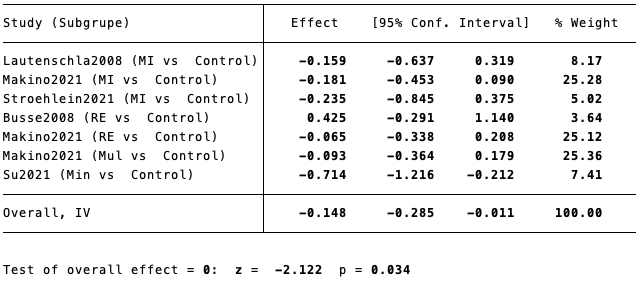


*Note*: MI: Moderate-Intensity aerobic exercise, RE: Resistance exercise, Mul: Multicomponent exercise, Min: Mind-body exercise.

**7.3 pairwise analysis for verbal fluency**

Fig 5. Forest plot of pairwise meta-analysis for verbal fluency

*Note*: MI: Moderate-Intensity aerobic exercise, RE: Resistance exercise, Mul: Multicomponent exercise.

Table 8. Pairwise meta-analysis of effectiveness for verbal fluency


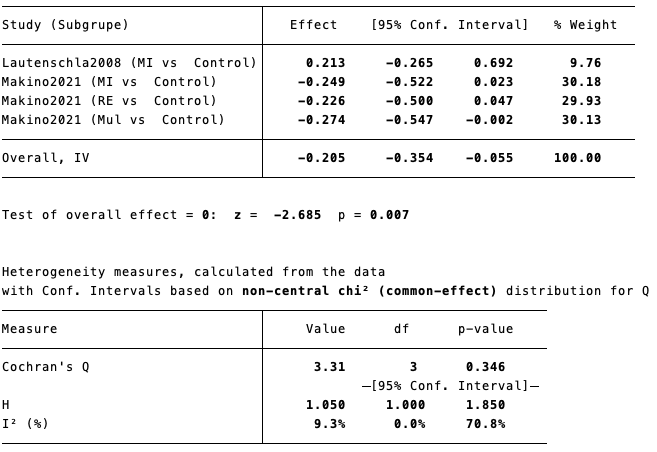


*Note*: MI: Moderate-Intensity aerobic exercise, RE: Resistance exercise, Mul: Multicomponent exercise.

**7.4 pairwise analysis for global cognitive function**

Fig 6. Forest plot of pairwise meta-analysis for global cognitive function

*Note*: MI: Moderate-Intensity aerobic exercise, HI: High-Intensity aerobic exercise, RE: Resistance exercise, Mul: Multicomponent exercise.

Table 9. Pairwise meta-analysis of effectiveness for global cognitive function


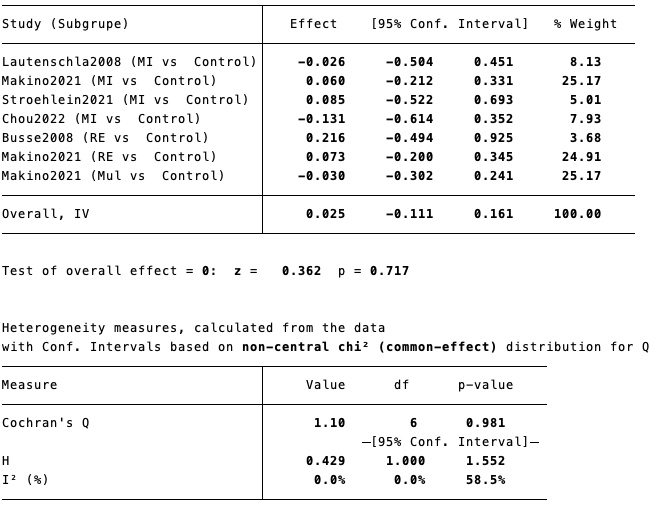


*Note*: MI: Moderate-Intensity aerobic exercise, HI: High-Intensity aerobic exercise, RE: Resistance exercise, Mul: Multicomponent exercise.

**7.5 pairwise analysis for attention**

Fig 7. Forest plot of pairwise meta-analysis for verbal fluency

*Note*: MI: Moderate-Intensity aerobic exercise, RE: Resistance exercise, Mul: Multicomponent exercise.

Table 10. Pairwise meta-analysis of effectiveness for verbal fluency


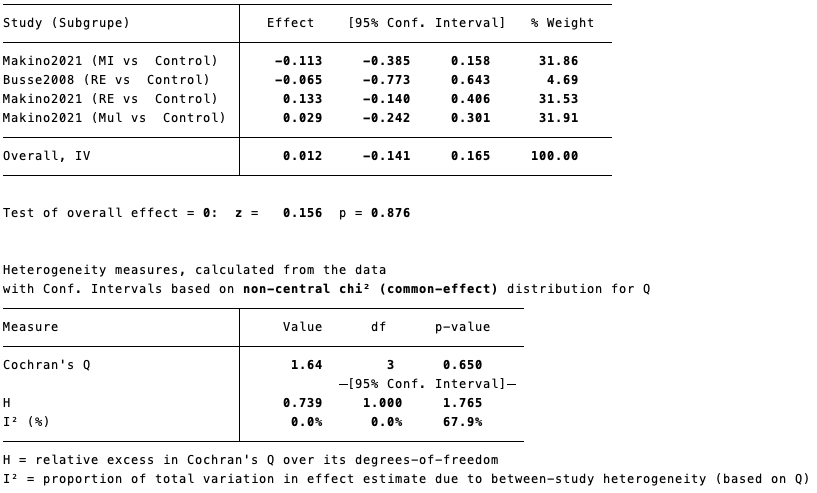


*Note*: MI: Moderate-Intensity aerobic exercise, RE: Resistance exercise, Mul: Multicomponent exercise.

# Appendix 8. Network meta-analysis

**8.1 Network analysis for memory**

**8.1.1 Global inconsistency test**

χ^2^ =8.74, *p* =0.07

No evidence for the existence of significant global inconsistency

**8.1.2 Local inconsistency test**


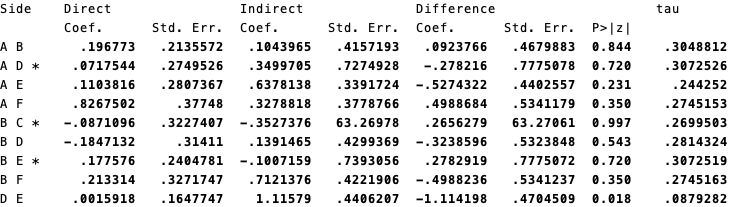


Fig 8. Local inconsistency test for memory

*Note:* A=Control, B=Moderate-Intensity aerobic exercise, C=High-Intensity aerobic exercise, D=Resistance exercise, E=Multicomponent exercise, F=Mind-body exercise.

**8.1.3 Loop inconsistency test**


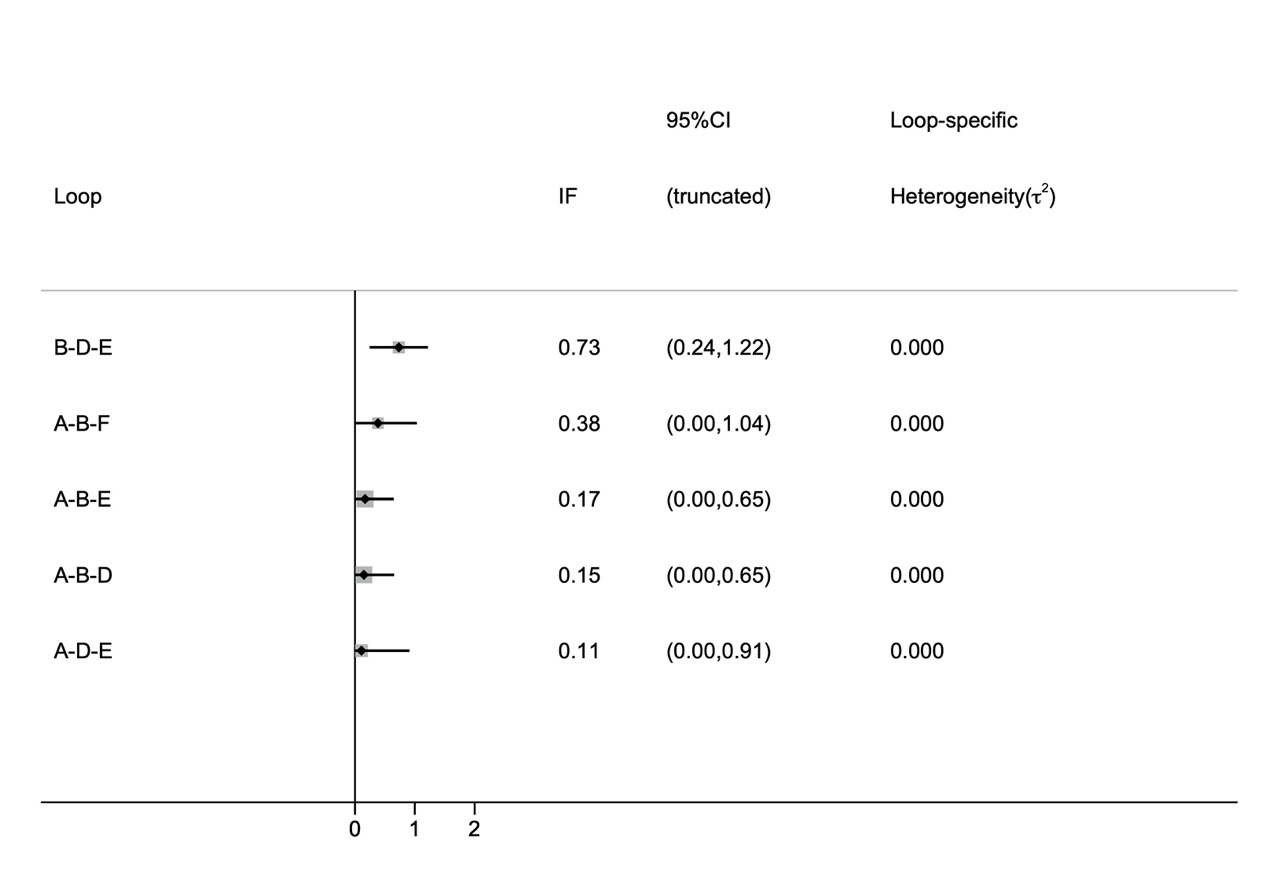


Fig 9. Loop inconsistency test for memory

*Note:* A=Control, B=Moderate-Intensity aerobic exercise, C=High-Intensity aerobic exercise, D=Resistance exercise, E=Multicomponent exercise, F=Mind-body exercise.

**8.1.4 Contribution plot for memory**


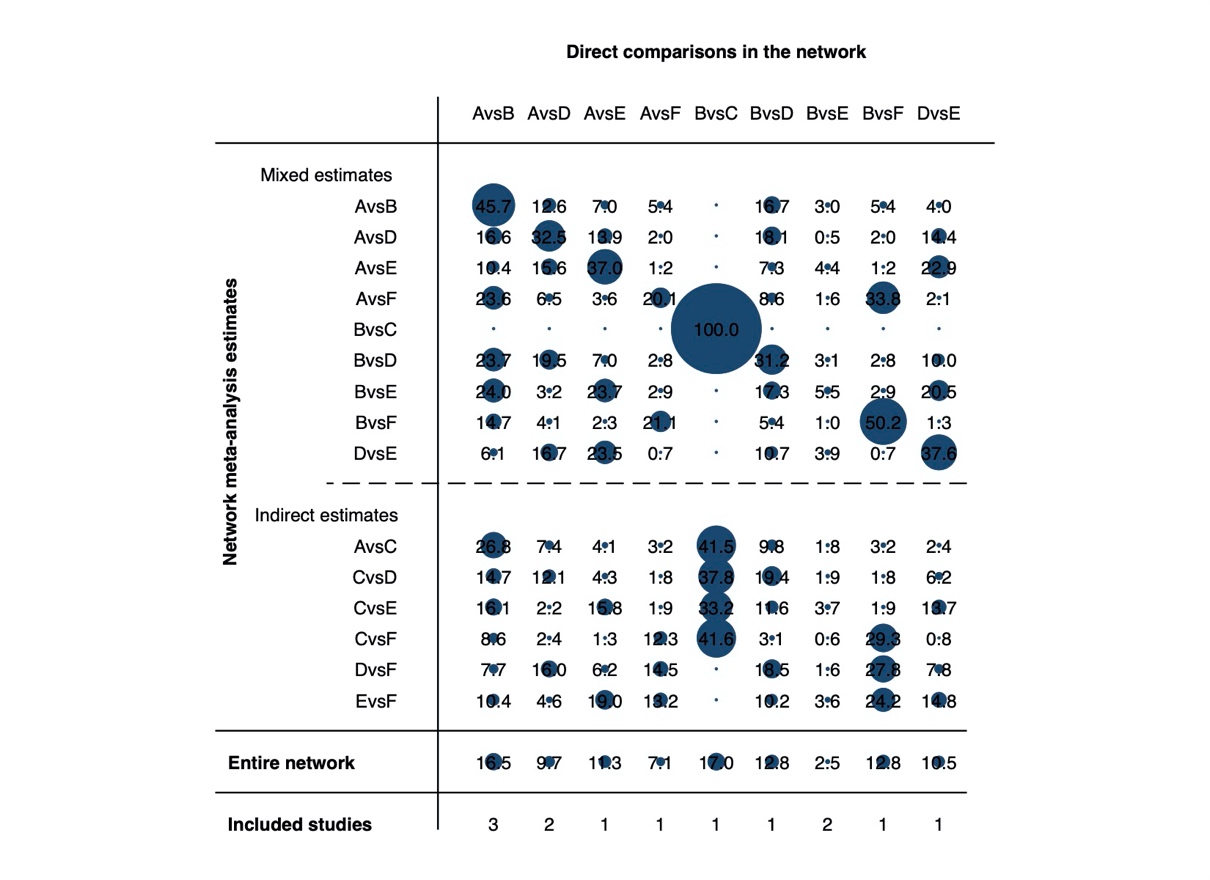


Fig 10. Contribution plot for memory

*Note:* A=Control, B=Moderate-Intensity aerobic exercise, C=High-Intensity aerobic exercise, D=Resistance exercise, E=Multicomponent exercise, F=Mind-body exercise.

**8.2 Network analysis for executive function**

**8.2.1 Global inconsistency test**

χ^2^ =4.10, *p* =0.39

No evidence for the existence of significant global inconsistency

**8.2.2 Local inconsistency test**


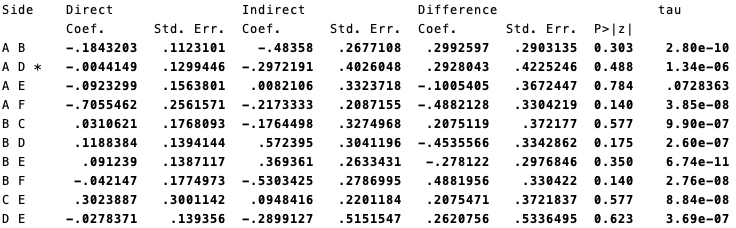


Fig 11. Local inconsistency test for executive function

*Note:* A=Control, B=Moderate-Intensity aerobic exercise, C=High-Intensity aerobic exercise, D=Resistance exercise, E=Multicomponent exercise, F=Mind-body exercise.

**8.2.3 Loop inconsistency test**

**
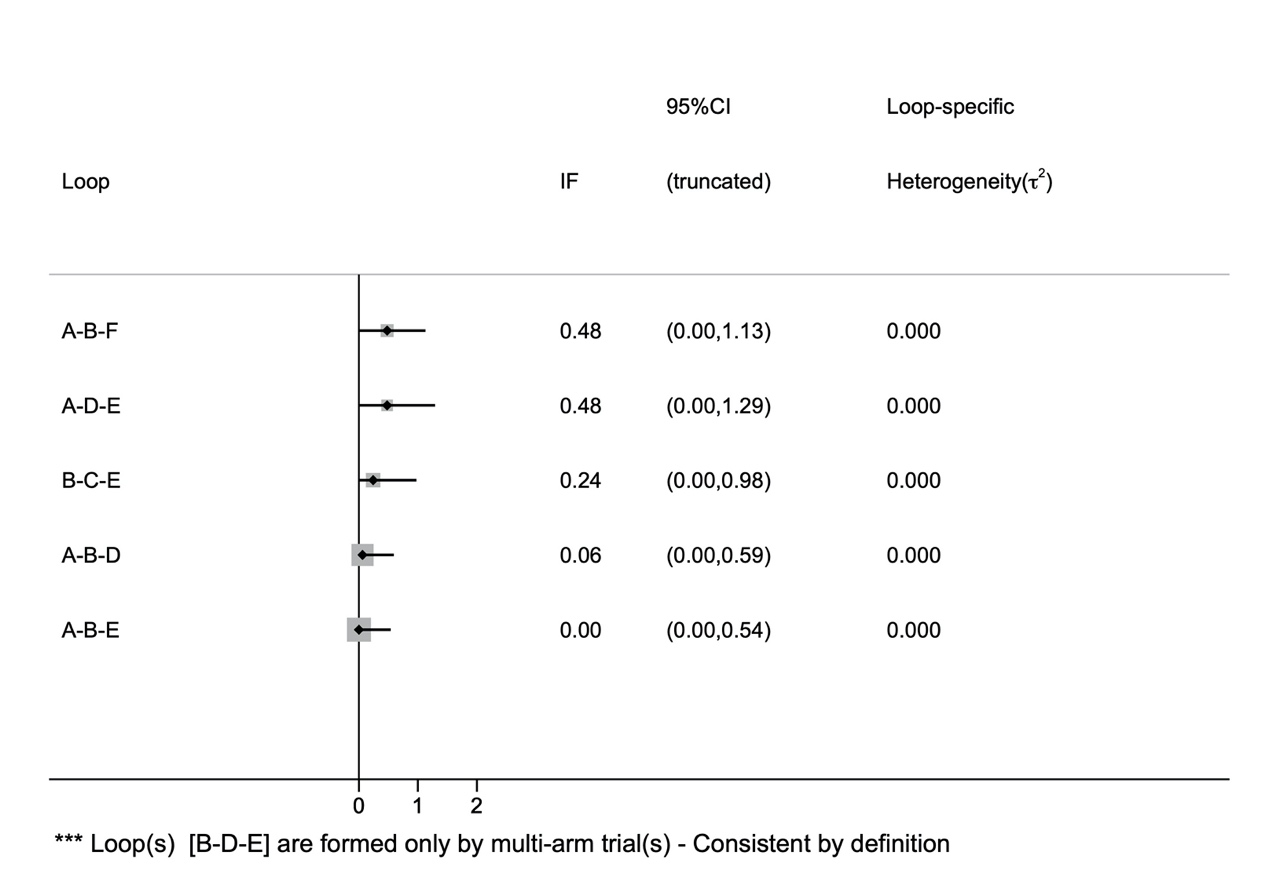
**

Fig 12. Loop inconsistency test for executive function

*Note:* A=Control, B=Moderate-Intensity aerobic exercise, C=High-Intensity aerobic exercise, D=Resistance exercise, E=Multicomponent exercise, F=Mind-body exercise.

**8.2.4 Contribution plot for executive function**

**
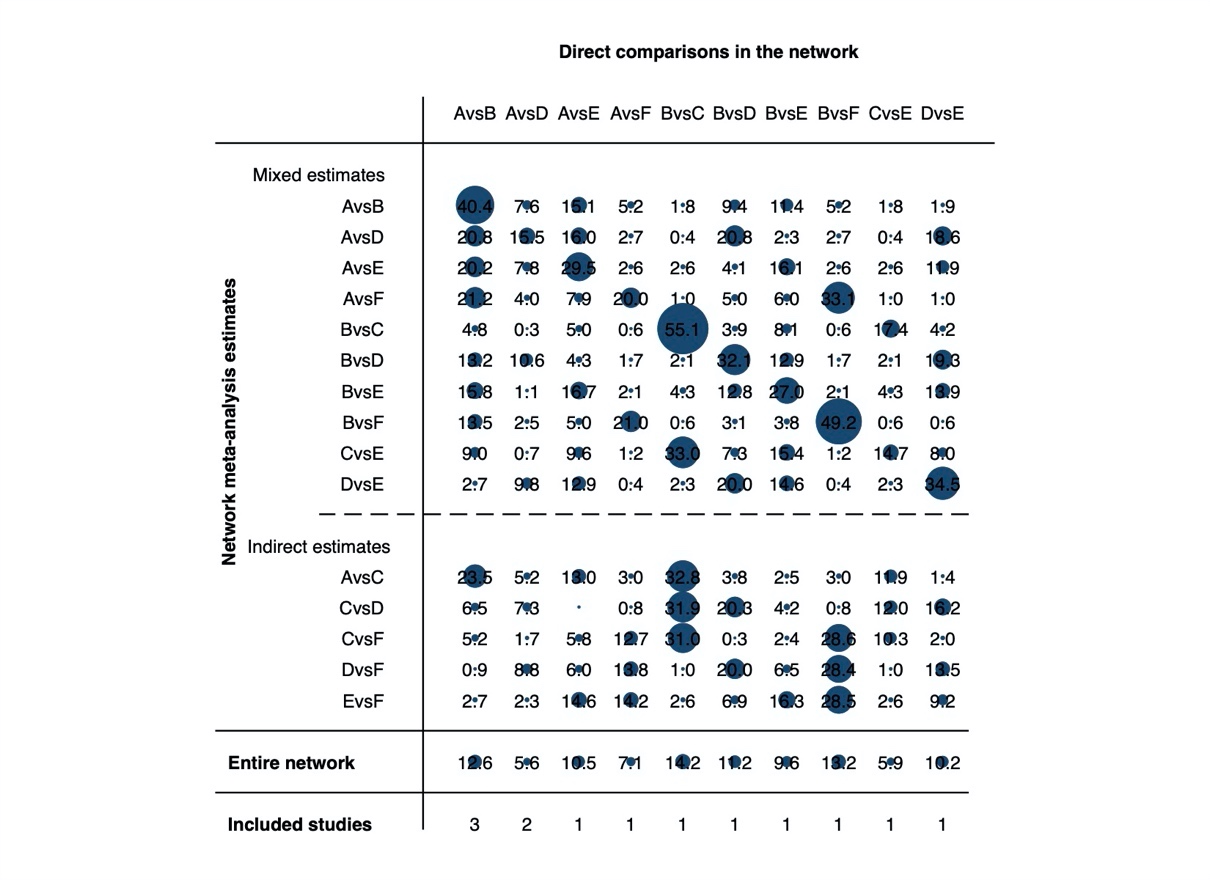
**

Fig 13. Contribution plot for executive function

*Note:* A=Control, B=Moderate-Intensity aerobic exercise, C=High-Intensity aerobic exercise, D=Resistance exercise, E=Multicomponent exercise, F=Mind-body exercise.

**8.3 Network analysis for verbal fluency**

**8.3.1 Global inconsistency test**

χ^2^ =2.71, *p* =0.10

No evidence for the existence of significant global inconsistency

**8.3.2 Local inconsistency test**


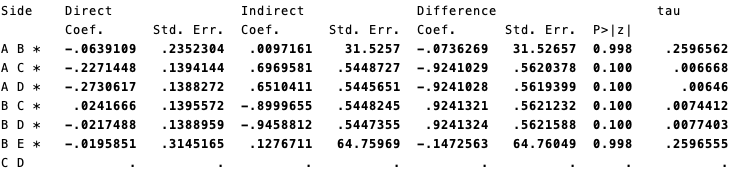


Fig 14. Local inconsistency test for verbal fluency

*Note:* A=Control, B=Moderate-Intensity aerobic exercise, C=Resistance exercise, D=Multicomponent exercise, E=Mind-body exercise.

**8.3.3 Loop inconsistency test**

**
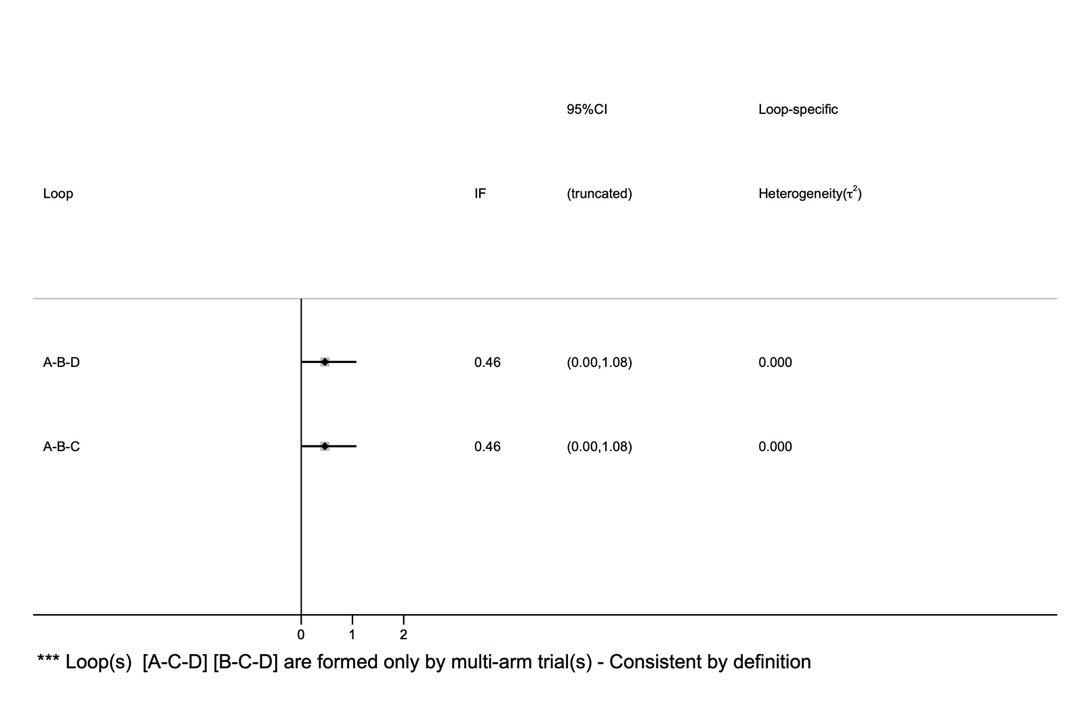
**

Fig 15. Loop inconsistency test for verbal fluency

*Note:* A=Control, B=Moderate-Intensity aerobic exercise, C=Resistance exercise, D=Multicomponent exercise, E=Mind-body exercise.

**8.3.4 Contribution plot for verbal fluency**

**
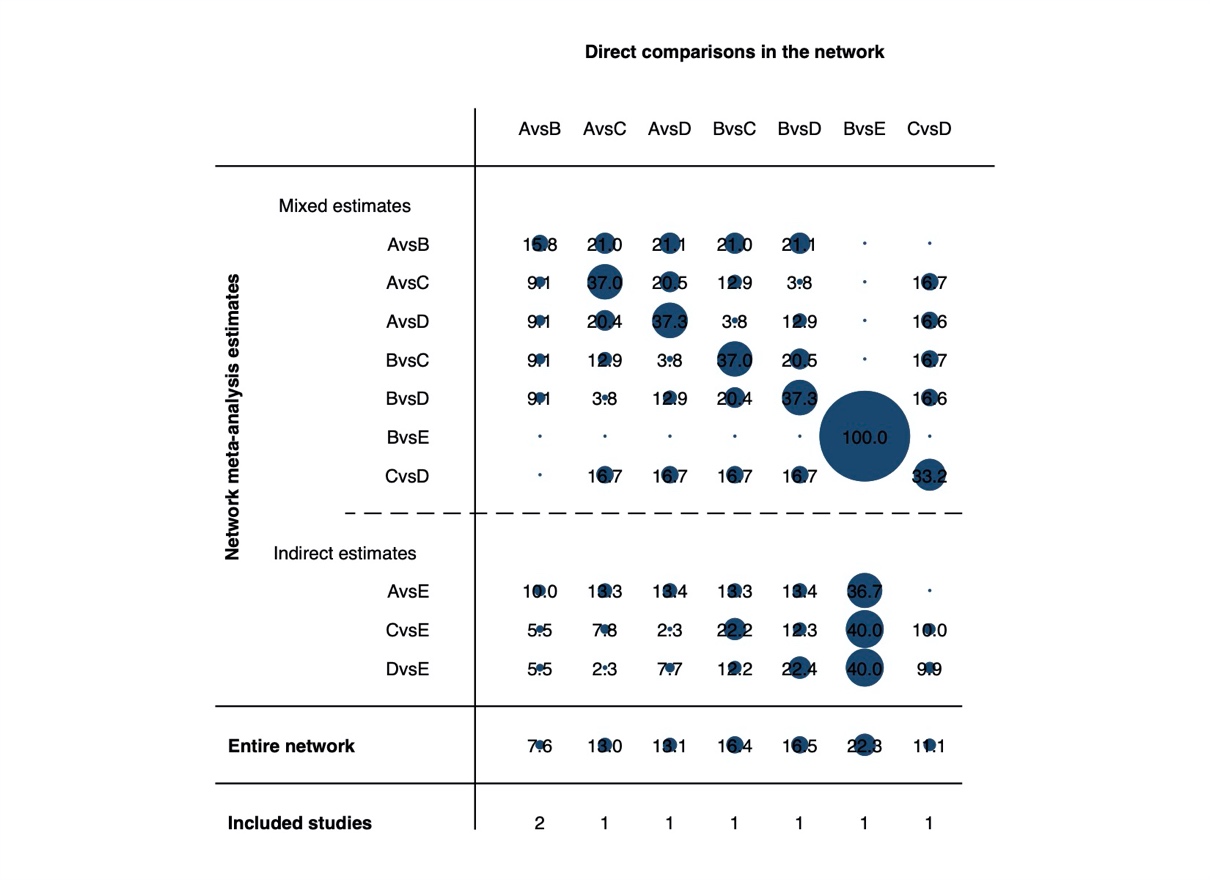
**

Fig 16. Contribution plot for verbal fluency

*Note:* A=Control, B=Moderate-Intensity aerobic exercise, C=Resistance exercise, D=Multicomponent exercise, E=Mind-body exercise.

**8.3.5** Table 11. the verbal fluency rankings for different types of exercise.

| Intervention | Verbal fluency | | |
| --- | --- | --- | --- |
|  | SUCRA | Mean rank | P (%) |
| Control | 12.4 | 4.5 | 0.6 |
| MI | 50.4 | 3.0 | 7.8 |
| RE | 59.5 | 2.6 | 31.1 |
| ME | 72.5 | 2.1 | 39.5 |
| MBE | 55.2 | 2.8 | 20.9 |

Notes: MI = Moderate-Intensity aerobic exercise; HI = High-Intensity aerobic exercise; RE = Resistance exercise; MBE = Mind-body exercise.

**8.4 Network analysis for attention**

**8.4.1 Global inconsistency test**

χ^2^ =0.26, *p* =0.61

No evidence for the existence of significant global inconsistency

**8.4.2 Local inconsistency test**


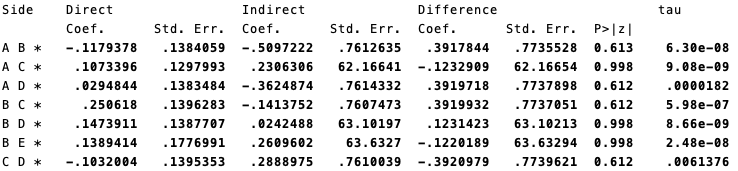


Fig 17. Local inconsistency test for attention

*Note:* A=Control, B=Moderate-Intensity aerobic exercise, C=Resistance exercise, D=Multicomponent exercise, E=Mind-body exercise.

**8.4.3 Loop inconsistency test**

**
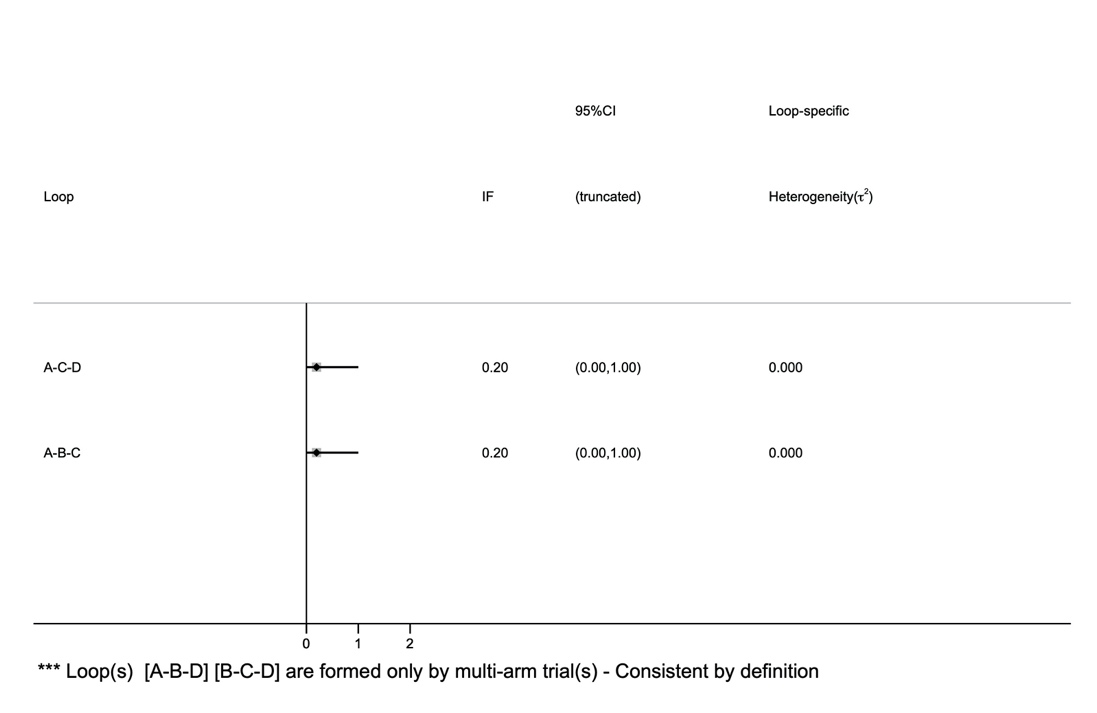
**

Fig 18. Loop inconsistency test for attention

*Note:* A=Control, B=Moderate-Intensity aerobic exercise, C=Resistance exercise, D=Multicomponent exercise, E=Mind-body exercise.

**8.4.4 Contribution plot for attention**

**
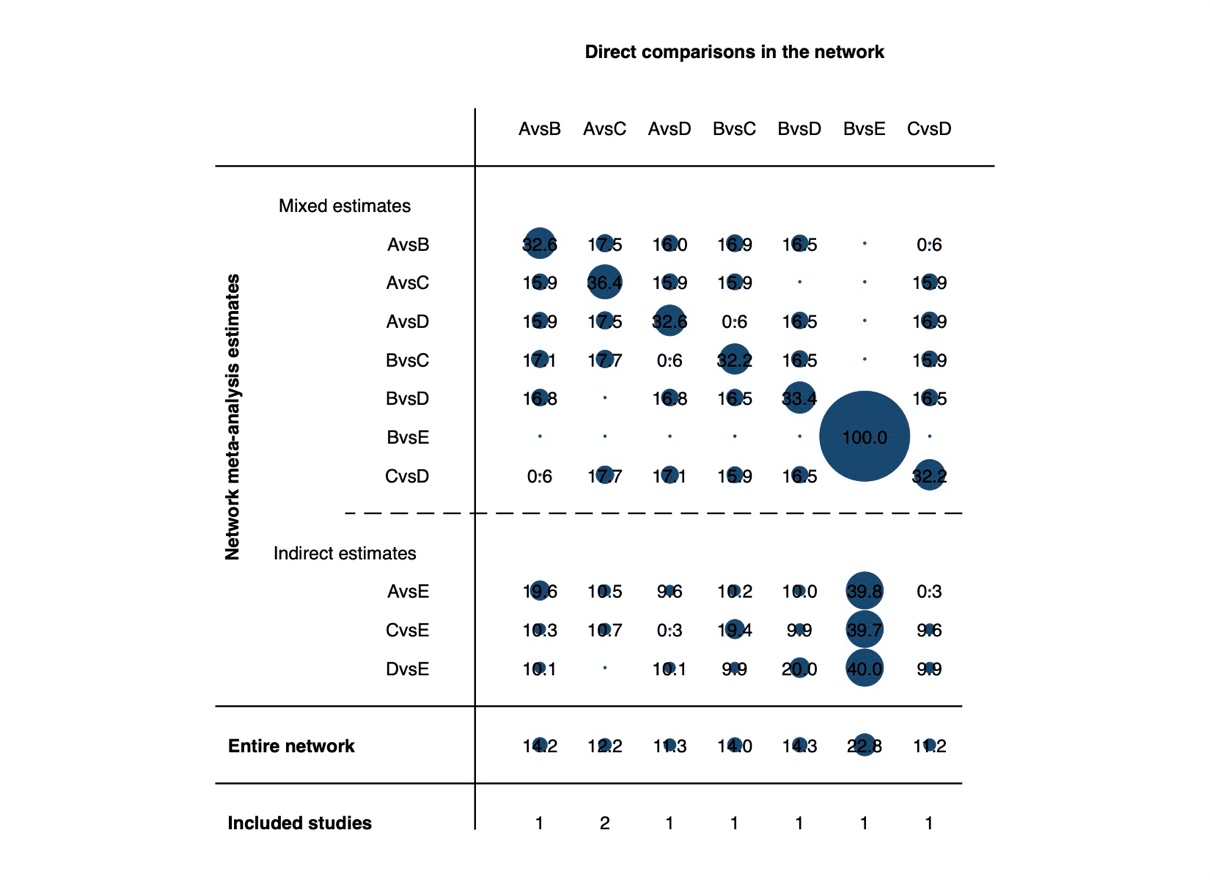
**

Fig 19. Contribution plot for attention

*Note:* A=Control, B=Moderate-Intensity aerobic exercise, C=Resistance exercise, D=Multicomponent exercise, E=Mind-body exercise.

**8.4.5** Table 12. the attention function rankings for different types of exercise.

| Intervention | Attention | | |
| --- | --- | --- | --- |
|  | SUCRA | Mean rank | P (%) |
| Control | 49.6 | 3.0 | 9.8 |
| MI | 14.4 | 4.4 | 0.3 |
| RE | 80.0 | 1.8 | 49.3 |
| ME | 53.8 | 2.8 | 26.5 |
| MBE | 52.2 | 2.9 | 14.1 |

Notes: MI = Moderate-Intensity aerobic exercise; HI = High-Intensity aerobic exercise; RE = Resistance exercise; MBE = Mind-body exercise.

**8.5 Network analysis for global cognitive function**

**8.5.1 Global inconsistency test**

χ^2^ =4.45, *p* =0.22

No evidence for the existence of significant global inconsistency

**8.5.2 Local inconsistency test**


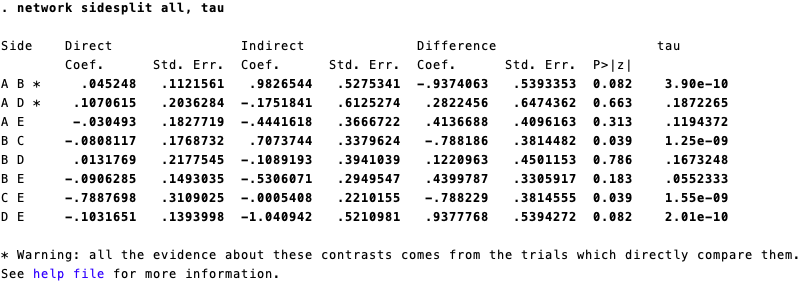


Fig 20. Local inconsistency test for global cognitive function

*Note:* A=Control, B=Moderate-Intensity aerobic exercise, C=High-Intensity aerobic exercise, D=Resistance exercise, E=Multicomponent exercise.

**8.5.3 Loop inconsistency test**

**
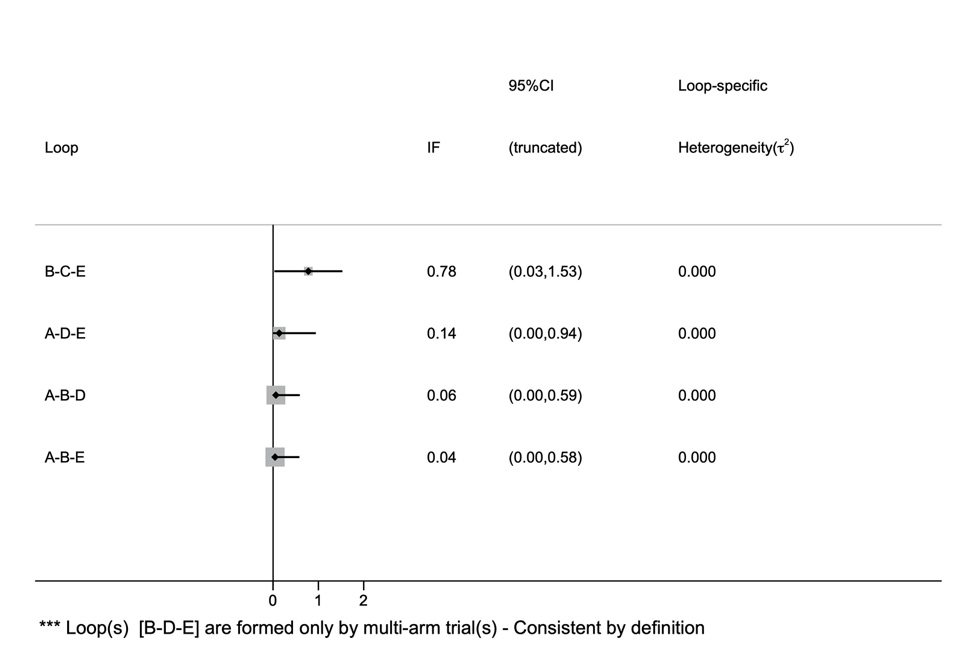
**

Fig 21. Loop inconsistency test for global cognitive function

*Note:* A=Control, B=Moderate-Intensity aerobic exercise, C=High-Intensity aerobic exercise, D=Resistance exercise, E=Multicomponent exercise.

**8.5.4 Contribution plot for global cognitive function**

**
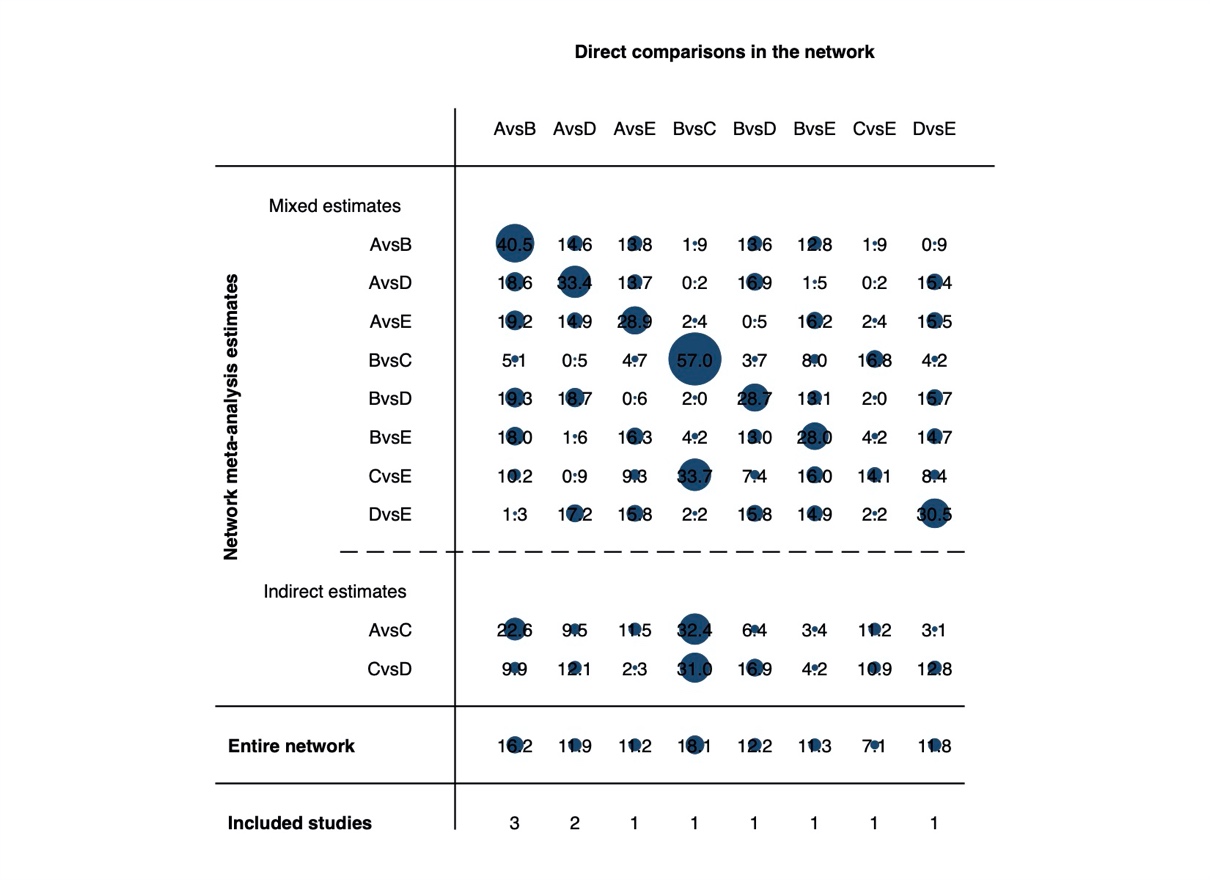
**

Fig 22. Contribution plot for global cognitive function

*Note:* A=Control, B=Moderate-Intensity aerobic exercise, C=High-Intensity aerobic exercise, D=Resistance exercise, E=Multicomponent exercise.

**8.5.5** Table 13. the global cognition rankings for different types of exercise.

| Intervention | Global cognition | | |
| --- | --- | --- | --- |
|  | SUCRA | Mean rank | P (%) |
| Control | 37.6 | 3.5 | 5.6 |
| MI | 61.4 | 2.5 | 19.6 |
| HI | 77.4 | 1.9 | 58.4 |
| RE | 57.3 | 2.7 | 13.7 |
| ME | 16.4 | 4.3 | 2.6 |

Notes: MI = Moderate-Intensity aerobic exercise; HI = High-Intensity aerobic exercise; RE = Resistance exercise; ME=Multicomponent exercise;

# Appendix 9. Regression analysis


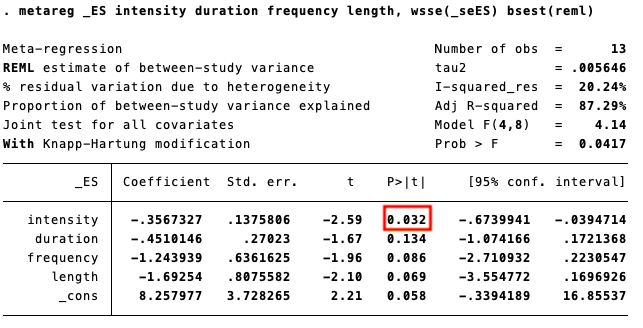


Fig 23. Meta-regression analyses for memory

# Appendix 10. Sensitivity analysis

**10.1 Sensitivity analysis for executive function**


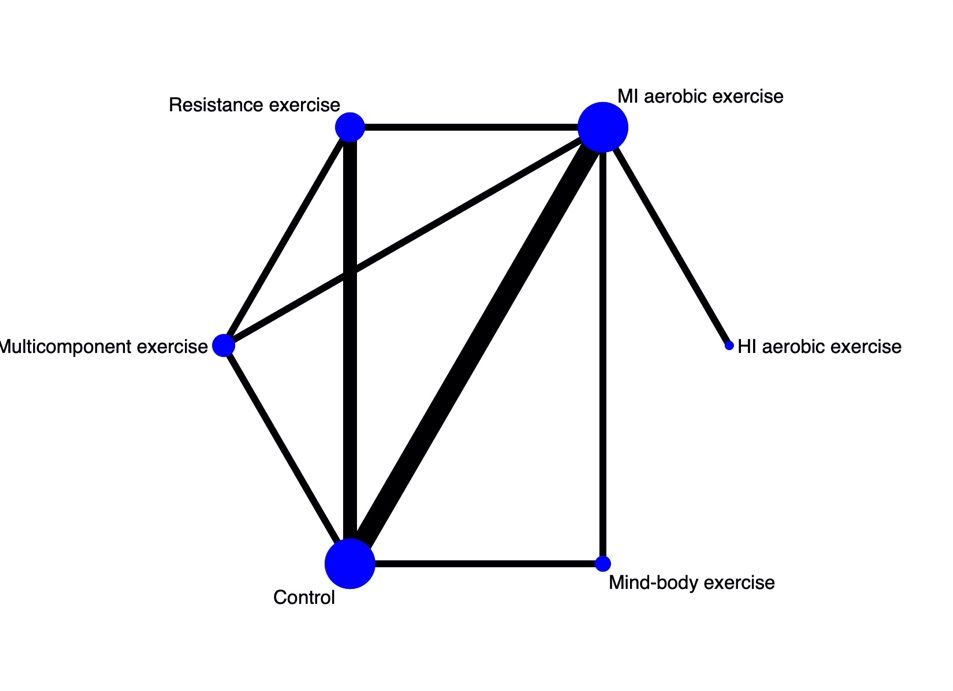


Fig 24. Network plot for executive function (sensitivity analysis)

*Note:* MI aerobic exercise: Moderate-Intensity aerobic exercise, HI aerobic exercise: High-Intensity aerobic exercise.


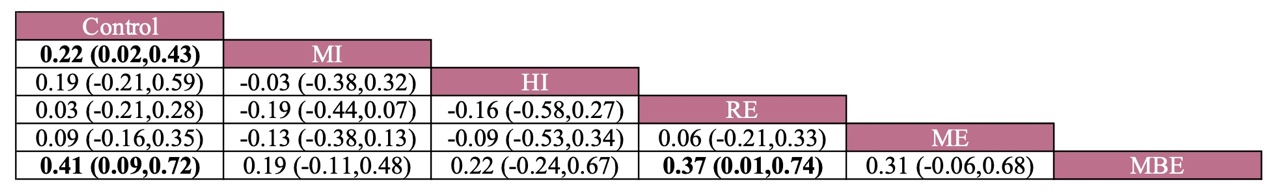


Fig 25. Effectiveness comparison for executive function (sensitivity analysis)

*Note:* MI: Moderate-Intensity aerobic exercise, HI: High -Intensity aerobic exercise, RE: Resistance exercise, ME: Multicomponent exercise, MBE: Mind-body exercise.

Fig 26. Cumulative ranking probability plot for executive function (sensitivity analysis)

*Note:* MI aerobic exercise: Moderate-Intensity aerobic exercise, HI aerobic exercise: High-Intensity aerobic exercise.

**10.2 Sensitivity analysis for global cognitive function**


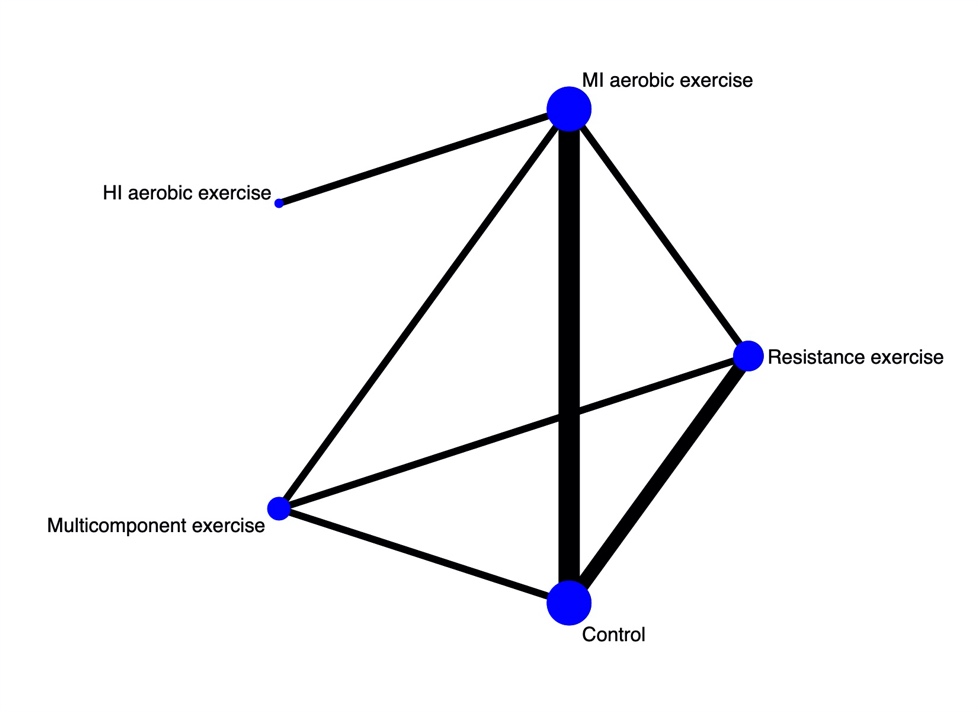


Fig 27. Network plot for global cognitive function (sensitivity analysis)

*Note:* MI aerobic exercise: Moderate-Intensity aerobic exercise, HI aerobic exercise: High-Intensity aerobic exercise.


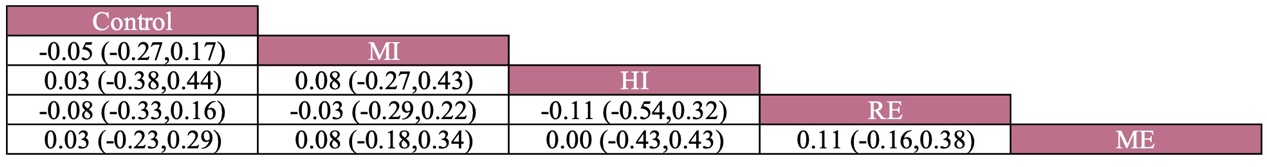


Fig 28. Effectiveness comparison for global cognitive function (sensitivity analysis)

*Note:* MI: Moderate-Intensity aerobic exercise, HI: High -Intensity aerobic exercise, RE: Resistance exercise, ME: Multicomponent exercise, MBE: Mind-body exercise.


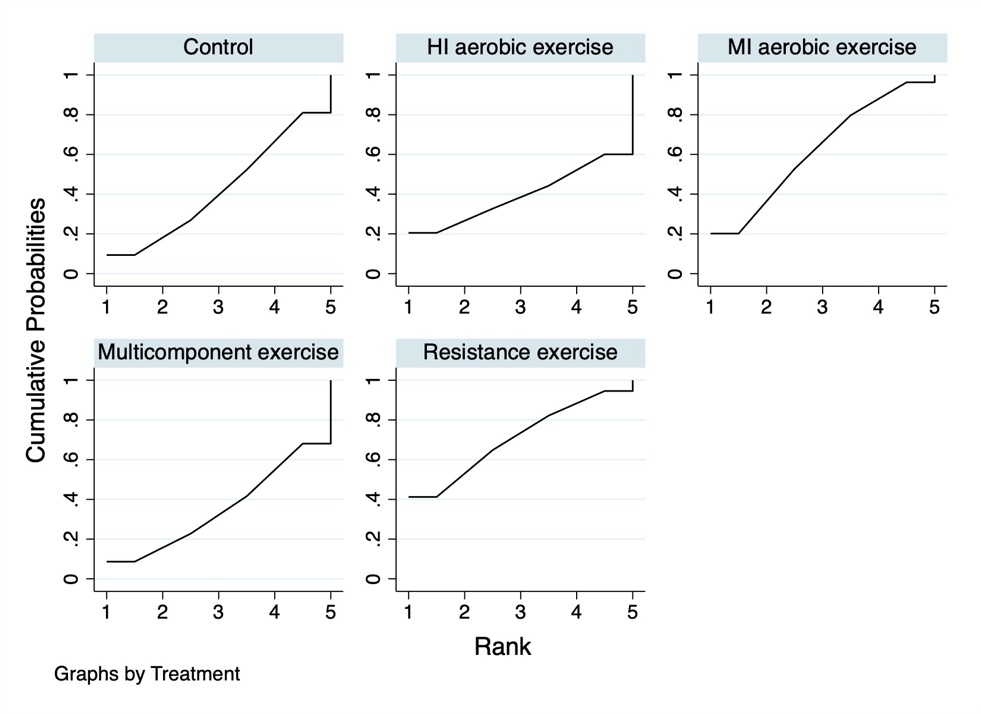


Fig 29. Cumulative ranking probability plot for global cognitive function (sensitivity analysis)

*Note:* MI aerobic exercise: Moderate-Intensity aerobic exercise, HI aerobic exercise: High-Intensity aerobic exercise.

# Appendix 11. Small study effects

**11.1 Small study effects** **test** **for memory**


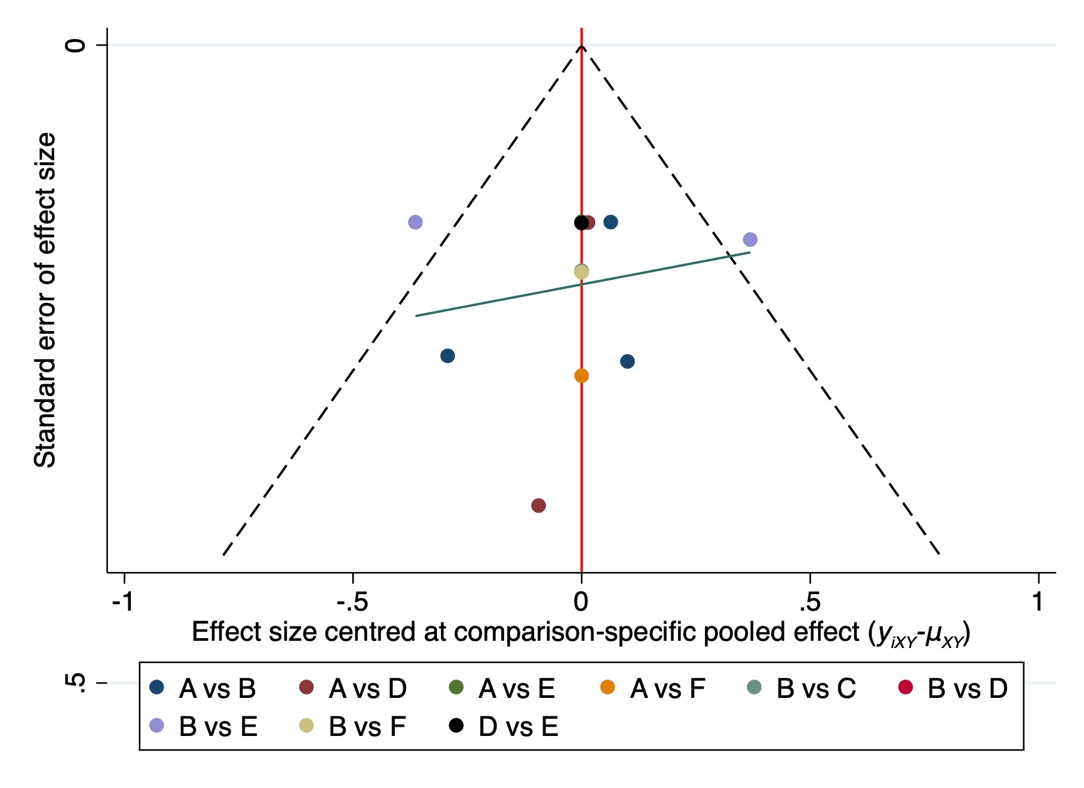


Fig 30. Funnel plot for memory

*Note:* A=Control, B=Moderate-Intensity aerobic exercise, C=High -Intensity aerobic exercise, D=Resistance exercise, E=Multicomponent exercise, F=Mind-body exercise.


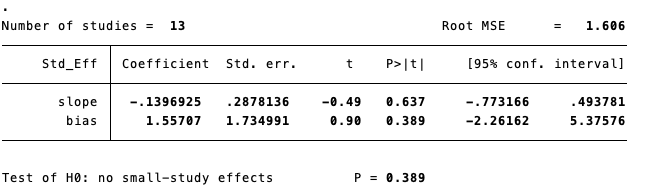


Fig 31. Egger's test for memory

**11.2 Small study effects** **test for executive function**


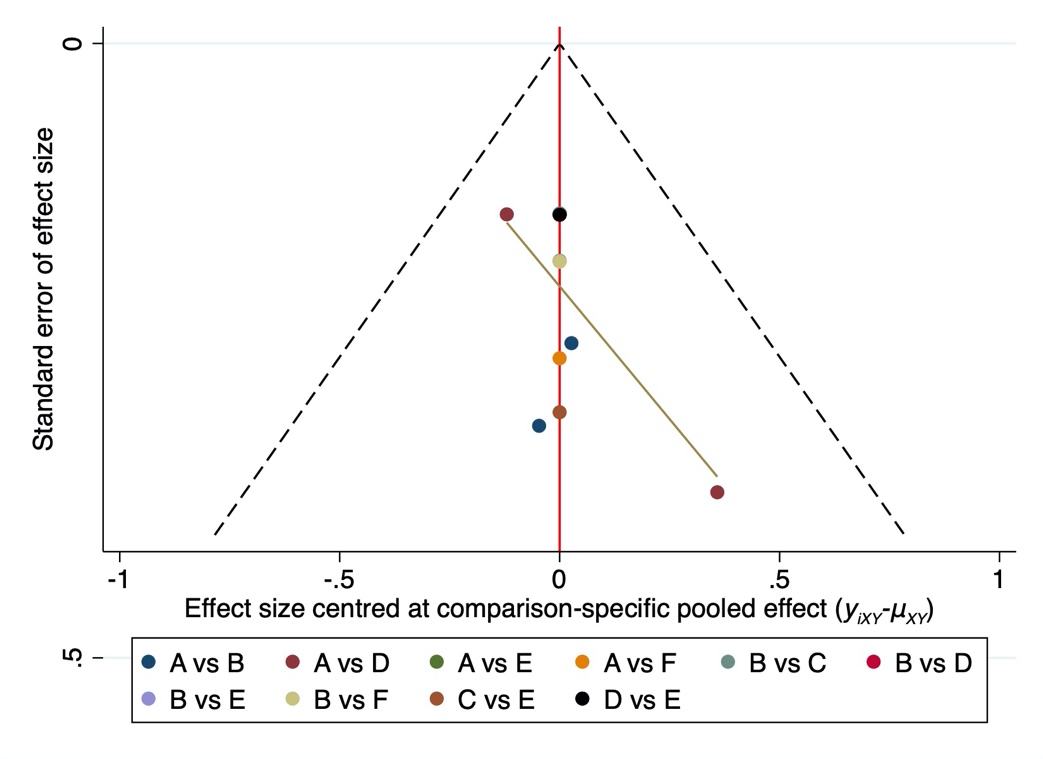


Fig 32. Funnel plot for executive function

*Note:* A=Control, B=Moderate-Intensity aerobic exercise, C=High -Intensity aerobic exercise, D=Resistance exercise, E=Multicomponent exercise, F=Mind-body exercise.


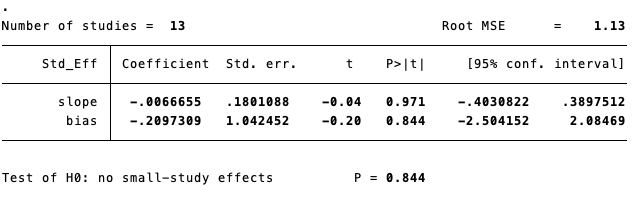


Fig 33. Egger's test for executive function

**11.3 Small study effects** **test for verbal fluency**


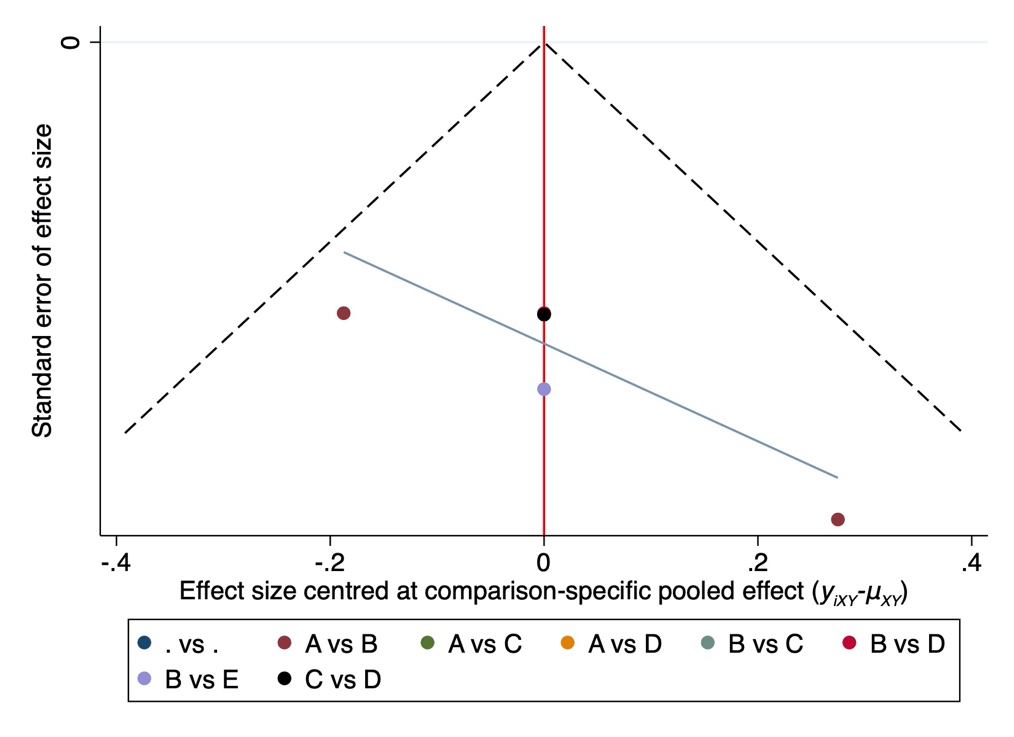


Fig 34. Funnel plot for verbal fluency

*Note:* A=Control, B=Moderate-Intensity aerobic exercise, C=Resistance exercise, D=Multicomponent exercise, E=Mind-body exercise.


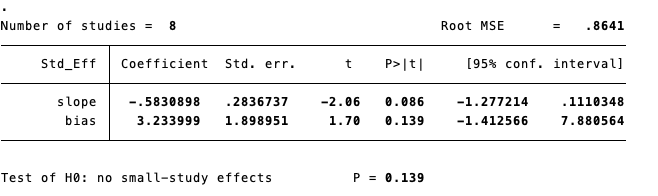


Fig 35. Egger's test for verbal fluency

**11.4 Small study effects** **test for attention**


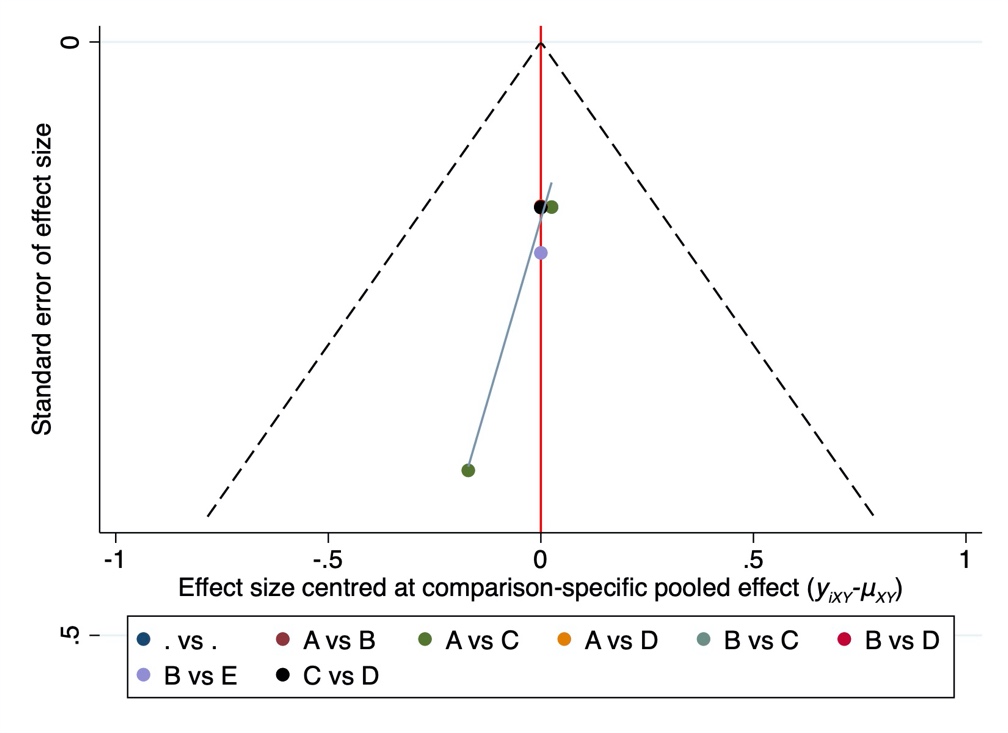


Fig 36. Funnel plot for attention

*Note:* A=Control, B=Moderate-Intensity aerobic exercise, C=Resistance exercise, D=Multicomponent exercise, E=Mind-body exercise.


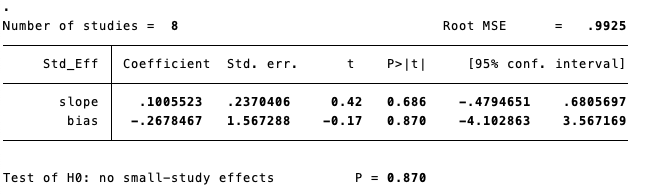


Fig 37. Egger's test for attention

**11.5 Small study effects** **test for global cognitive function**


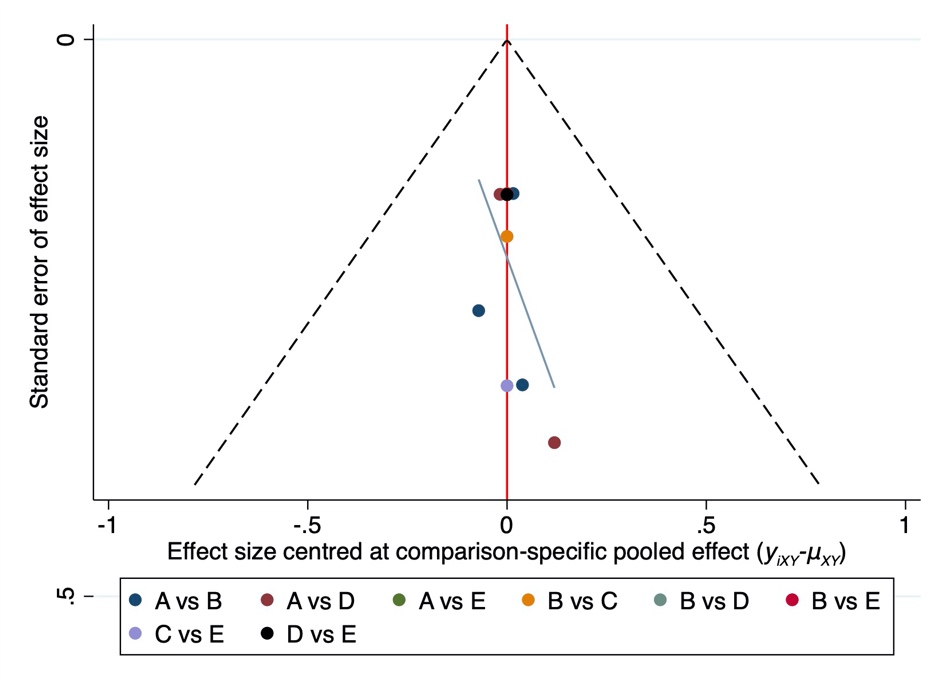


Fig 38. Funnel plot for global cognitive function

*Note:* A=Control, B=Moderate-Intensity aerobic exercise, C=High -Intensity aerobic exercise, D=Resistance exercise, E=Multicomponent exercise.


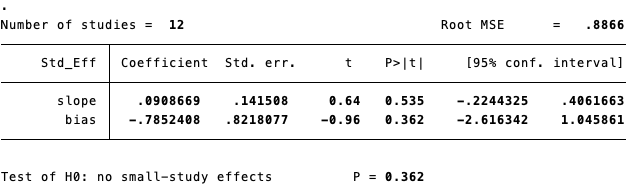


Fig 39. Egger's test for global cognitive function
